# Exact solutions and equi-dosing regimen regions for multi-dose pharmacokinetics models with transit compartments

**DOI:** 10.1007/s10928-020-09719-8

**Published:** 2020-10-10

**Authors:** F. Hof, L. J. Bridge

**Affiliations:** 1 Swansea University, Swansea, UK; 2 Medical University of Vienna, Vienna, Austria; 3 Department of Engineering Design and Mathematics, University of the West of England, Bristol, UK

**Keywords:** Mathematical pharmacology, Pharmacokinetics, Compartment models, Differential equations, Transit compartments, Regimen design

## Abstract

Compartmental models which yield linear ordinary differential equations (ODEs) provide common tools for pharmacokinetics (PK) analysis, with exact solutions for drug levels or concentrations readily obtainable for low-dimensional compartment models. Exact solutions enable valuable insights and further analysis of these systems. Transit compartment models are a popular semi-mechanistic approach for generalising simple PK models to allow for delayed kinetics, but computing exact solutions for multi-dosing inputs to transit compartment systems leading to different final compartments is nontrivial. Here, we find exact solutions for drug levels as functions of time throughout a linear transit compartment cascade followed by an absorption compartment and a central blood compartment, for the general case of *n* transit compartments and *M* equi-bolus doses to the first compartment. We further show the utility of exact solutions to PK ODE models in finding constraints on equi-dosing regimen parameters imposed by a prescribed therapeutic range. This leads to the construction of equi-dosing regimen regions (EDRRs), providing new, novel visualisations which summarise the safe and effective dosing parameter space. EDRRs are computed for classical and transit compartment models with two- and three-dimensional parameter spaces, and are proposed as useful graphical tools for informing drug dosing regimen design.

## Introduction

Mathematical models for the absorption, distribution and elimination of drugs are common in the pharmacokinetics (PK) literature. Typically, a drug’s route through the body to its pharmacological effect site is modelled as a number of compartments, with transfer between compartments being governed by pharmacokinetic rate laws. It is common to consider only one or two compartments, with linear pharmacokinetics, resulting in low-dimensional linear ordinary differential equation (ODE) systems. However, such models are not sufficient to capture delay-type effects, whereby some time passes before the drug appears at measurable levels in the systemic circulation [44]. If a significant “drug absorption delay” [44, 46] is observed, then a lag-time is sometimes introduced into solutions to the simple models to account for the delay, while avoiding any mechanistic considerations of the underlying delay roaaataaat esses. This simple approach may be used to paramaterise a system delay, but it is known that absorption delay is a complex process that is not switch-like. As such, lag-time models can give a poor characterisation of absorption-phase PK.

Transit compartment models have been proposed to capture delay effects in PK time courses, through a semi-mechanistic approach of increasing the number of compartments through which the drug is transferred en route to the central compartaament (blood) [26, 27, 32, 33, 44, 46, 47]. The development of “full” or accurate mechanistic physiologically-based PK models requires much experimental data and knowledge which may be unavailable. For systems exhibiting delays, transit compartment models therefore represent a physiologically plausible and mathematically practical alternative to the change-point approach of lag-time models.

While transit compartment models add additional complexity beyond one- or two-compartment models, their outputs in response to a single bolus dose to the first compartment may be derived analytically in certain cases. An analytical solution permits relatively straightforward approaches to both sensitivity analysis (particularly when varying the number of compartments) and model fitting. In [44], the response to a bolus dose is considered for an *n*-transit compartment model with an additional “absorption compartment” between transit compartment *n* and the central circulation. An analytical solution for the drug concentration in the *n*th transit compartment is presented, and used as an input to the absorption compartment ODE, together with the Stirling approximation, to transform a discrete optimisation problem to a continuous one for the purpose of data fitting. The analysis and parameter estimation is limited to the case of single bolus dose, and exact solutions are not found for the absorption and central compartments. Further mathematical properties for transit compartment models in pharmacodynamics are presented in [55].

Drug dosing regimens often use multi-dosing treatments, whereby a regular dose is given at regular specified dosing intervals. For intravenous (IV) or oral administration of a drug, the analysis of a one- or two-compartment model with periodic bolus input yields analytical solutions for the drug level in the central compartment (e.g. [11, 24, 43, 45]). Time courses of drug level (or concentration) simulations show transient and steady-state (periodic) profiles which may then be compared with minimum effective and maximum safe levels which define a therapeutic window. We will introduce the idea of an equi-dosing regimen region of (dose,interval)-space, which gives a guide for selecting therapeutic equi-dosing regimens.

Given that it is accepted that drug absorption delay may be a significant pharmacokinetic effect, analysis of transit compartment models, incorporating multi-dose inputs, appears to be a valuable pursuit. Some attention has been paid to this problem in the PK literature. Shen et al. [46] extend the work of [44] to derive a solution to the multi-dose problem at the *n*th transit compartment using the method of superposition. However, the challenge remains to solve for the drug level in the central compartment exactly, and to use this result as a platform for further analysis, including design of safe dosing regimens.

In this paper, we present new mathematical and graphical results which both generalise transit compartment PK models and summarise dosing regimen constraints given by therapeutic ranges imposed on these models. In ‘Methods: multi-dosing models with and without transit compartments—formulation’, we formulate linear ordinary differential equation (ODE) models for one-compartment, two-compartment and transit compartment pharmacokinetics, extending the work of [44] to consider drug level in the central compartment. In ‘Analytical solutions for equi-dosing regimens’, we review standard analytical results for multi-bolus and multi-infusion dosing, and derive a new exact solution for the transit compartment model with general number of compartments and doses. This new generalised solution and its improvement over existing models comprise our first main contribution. In ‘Results: equi-dosing time courses’, we present simulations and data fitting using the new analytical solutions, illustrating their predictive capability, and highlighting the error between the new exact solution and Stirling approximation solution of [44] for a single bolus dose. In ‘Results: equi-dosing regimen regions’, we present our second main contribution, namely the new concept of equi-dosing regimen regions (EDRRs), which provide a novel visualisation to summarise constraints on dosing regimen parameters. We conclude in ‘Discussion’ with a discussion of our main results, highlighting our contributions to the PK and mathematical modelling literature.

## Methods: multi-dosing models with and without transit compartments—formulation

### General compartmental model schematic

We use a compartmental approach to model a drug’s route from administration to the systemic circulation. Ultimately, the systemic circulation is treated as the final compartment in a cascade, hereafter referred to as the *central compartment*. The central compartment drug concentration (the drug amount per volume of distribution) is responsible for responses at drug effect sites [45], and we consider the drug level $a_{c}$ as the output in each of our models.

**Fig. 1:**
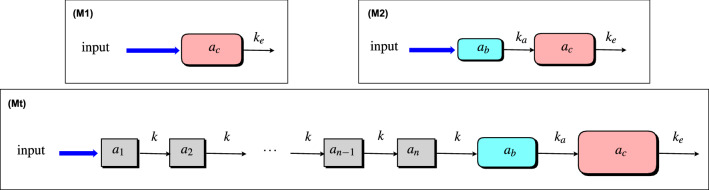
Compartmental model schematics. (M1) Single-compartment model—the input dose immediately appears in the central compartment, in amount $a_{c}$. (M2) Two-compartment model—the input dose first appears in an absorption compartment, in amount $a_{b}$. From here, it is transferred to the central compartment, in which the amount is $a_{c}$. Transfer from absorption to central compartment is a first order process, with rate constant $k_{a}$ (we consider $k_{a}> k_{e}$ [45])—and $k\ne k_{e},k_{a}$ [44]. (Mt) Transit-compartment model—the input dose first appears in the first of *n* transit compartments, which contain the amounts $a_{1}$, $a_{2}, \ldots , a_{n}$. Drug is transferred through the cascade of *n* transit compartments, as first order process with rate constant *k* for each compartment. From transit compartment *n*, drug is transferred to the absorption compartment. For all three models, the (first order) elimination rate constant is $k_{e}$

For intranvenous (IV) dosing, the drug immediately appears in the central compartment upon administration. We will refer to the corresponding model as a *single-compartment* or *one-compartment* model (Fig. 1, model (M1)). The amount of drug in the central compartment (the “drug level”) in this model is governed by an ordinary differential equation (ODE) which describes linear pharmacokinetics, whereby the drug is eliminated from the compartment as a first order process with elimination rate constant $k_{e}$. A two-compartment model (Fig. 1, model (M2)) in which drug appears in the central compartment via an absorption compartment is often used to model oral dosing [43, 45], where the absorption compartment is representative of the gastrointestinal (GI) tract. In response to a bolus dose input, the central compartment drug level $a_{c}$ in (M2) is both delayed and smoothed in comparison with the absorption compartment level $a_{b}$. In order to model a more pronounced delay by way of a semi-mechanistic compartmental schematic, we consider a transit compartment cascade feeding the absorption compartment, as in [44] (Fig. 1, model (Mt)). We note that such a modelling approach corresponds to the so-called *linear chain trick* [23].

### Dosing regimen inputs

**Fig. 2:**
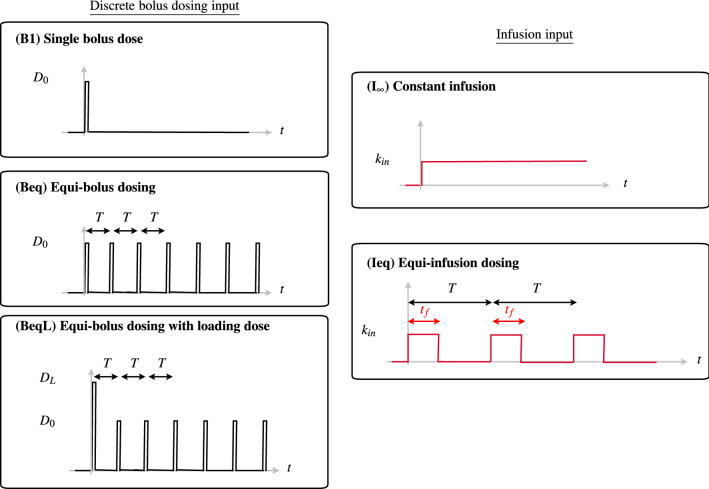
Dosing regimen input schematics. (B1) Single bolus dose $D_{0}$ (measured in mg) administered at time $t=0$. (Beq) Equi-bolus dosing, with a bolus dose $D_{0}$ mg administered at time $t=0$, and again at times $t=T,\; 2T,\; 3T$, etc. The dosing interval *T* is typically measured in hours. (BeqL) Equi-bolus dosing ($D_{0},T$) with an initial loading dose $D_{L}$ administered at time $t=0$. (I$_{\infty }$) Constant infusion, with drug infused into central compartment, starting at time $t=0$, at a rate $k_{in}$ (mg   h$^{-1}$). (Ieq) Equi-infusion dosing, with drug infused into central compartment at a rate $k_{in}$, periodically with period *T*. Each dosing interval infusion “on” for duration $t_{f}$, then infusion “off” for duration $(T-t_{f})$

Dosing patterns in therapeutics often consist of multi-dosing treatments, whereby doses are administered periodically [11, 43, 45, 46]. Models for multi-dosing are typically analysed under the assumption of *equi-dosing*, whereby both the dose $D_{0}$ and dosing interval (time between doses) *T* are constants. In this case, the couple $(T,D_{0})$ constitutes the *dosing regimen*. Here we principally investigate equi-dosing regimens in which the input is a fixed bolus dose administered periodically to the central, absorption or first transit compartment (see Fig. 2, regimen (Beq)). We also consider a simple perturbation to this regimen, where a *loading dose* $D_{L}$ (greater than $D_{0}$) is administered at $t=0$, followed by equi-dosing (Fig. 2, regimen (BeqL)). This regimen is common in practice, such that a loading dose helps to achieve therapeutic drug levels rapidly, while the subsequent equi-dosing *maintains* therapeutic levels [45].

We further consider the case of equi-infusion dosing, whereby for model (M1), the input is periodic constant infusions of drug to the central compartment, over fixed “on” time intervals, separated by fixed “off” intervals (Fig. 2, regimen (Ieq)).

### Model assumptions and considerations

Transit compartment models (TCMs) for PK typically take the drug amounts in each compartment ($a_{i}$ for $i=1, \ldots , n$, $a_{b}$ and $a_{c}$ in Fig. 1) as state variables (see, e.g., [26, 44, 46]). The bioavailability factor *F*, which is the fraction of drug dose ultimately absorbed into the systemic circulation, is an important consideration. Existing TCM models and simpler models introduce this “correction” factor at different points in the cascade [23, 26, 34, 43, 44, 46]. Here we follow [23, 43] in introducing the factor immediately, such that the first compartment in the cascade is fed by the *effective dose* ($F\times \text {dose}$).

We consider equi-dosing regimens for both IV infusion and *n*-transit compartment cascades, to allow analysis of drug level time course features in general. Together with prescribed *therapeutic ranges*, our analysis will indicate regions of equi-dosing regimen parameter space which give safe and effective treatments at steady-state. A therapeutic range is typically defined by minimum effective and maximum safe drug *concentrations* in the central compartment. For a given drug amount $a_{c}$, the corresponding drug concentration $C_{c}$ is given by $a_{c}/V$, where *V* is the calculated volume of the central compartment [25, 43, 45]. Therefore, for a fixed, known volume *V*, we can state a corresponding therapeutic range in terms of drug amounts, requiring

2.1 $$\begin{aligned} D_{me}< a_{c} < D_{MS}, \end{aligned}$$

where $D_{me}$ and $D_{MS}$ are the minimum drug level for therapeutic effect and maximum safe drug level respectively.

For the transit compartment model, the transit cascade prior to the absoprtion compartment consists of *n* compartments, each with an elimination rate *k*. The mean transit time (MTT) for this cascade is given by (see [44])

2.2 $$\begin{aligned} MTT=\frac{n}{k}. \end{aligned}$$

### Ordinary differential equation formulation

For a multi-dosing regimen, the problem can be stated as an initial value problem (IVP) for the state variables (e.g. $a_{i}$ for $i=1, \ldots , n$, $a_{b}$ and $a_{c}$ for model (Mt)) with impulsive drug inputs to the first compartment described by a dosing-rate forcing function comprising Dirac delta functions for discrete impulses (as in [26, 34]).

**Table 1:** Initial value problems (IVPs) of interest for single- and two-compartment models

Model	Input	IVP
M1	Beq	$${\begin{array}{ll} \frac{da_{c}}{dt} &{} = -k_{e}a_{c} + g_{B}(t), \\ a_{c}(0) &{} =0.\end{array}}$$
M1	BeqL	$${\begin{array}{ll} \frac{da_{c}}{dt} &{} = -k_{e}a_{c} + g_{BL}(t), \\ a_{c}(0) &{} =0.\end{array}}$$
M1	Ieq	$${\begin{array}{ll} \frac{da_{c}}{dt} &{} = -k_{e}a_{c} + g_{I}(t), \\ a_{c}(0) &{} =0.\end{array}}$$
M2	Beq	$${\begin{array}{ll} \frac{da_{b}}{dt} &{} = -k_{a}a_{b} + g_{B}(t), \\ \frac{da_{c}}{dt} &{} = k_{a}a_{b} -k_{e} a_{c}, \\ a_{b}(0) &{} =a_{c}(0)=0.\end{array}}$$
M2	BeqL	$${\begin{array}{ll} \frac{da_{b}}{dt} &{} = -k_{a}a_{b} + g_{BL}(t), \\ \frac{da_{c}}{dt} &{} = k_{a}a_{b} -k_{e} a_{c}, \\ a_{b}(0) &{} =a_{c}(0)=0.\end{array}}$$

Here, $\delta$ is the Dirac delta function, *M* is the number of doses given, and *T* is the dosing interval for bolus doses. Model and Input labels refer to Figs. 1 and 2

Firstly, the IVPs we consider for single-compartment IV multi-dosing and two-compartment oral multi-dosing are summarised in Table 1. For equi-bolus dosing with *M* doses $D_{0}$ at time intervals *T* starting at $t=0$ (regimen (Beq)), the input rate to the first compartment is

2.3 $$\begin{aligned} g_{B}(t)=\sum _{j=1}^{M}FD_{0}\delta (t-(j-1)T). \end{aligned}$$

If the first bolus dose is replaced with a larger loading dose $D_{L}$ (regimen (BeqL)), then the forcing rate function is

2.4 $$\begin{aligned} g_{BL}(t)=FD_{L}\delta (t)+\sum _{j=2}^{M}FD_{0}\delta (t-(j-1)T). \end{aligned}$$

For equi-infusion dosing (regimen Ieq) with infusion rate $k_{in}$, infusion “on” duration *T*, infusion “off” duration $t_{f}$, and *M* infusions, the forcing rate function is

2.5 $$\begin{aligned} g_{I}(t)=Fk_{in}\sum _{j=1}^{M}\big \{H(t-(j-1)T) - H(t-(j-1)T-t_{f})\big \}, \end{aligned}$$

where *H* is the Heaviside function.

We will consider solutions to the IVPs given in Table 1 in constructing the associated equi-dosing regimen regions. Beyond these relatively simple models, our analysis extends to the *n*-transit compartment model with input into the first transit compartment (model (Mt)). The governing equations consist of $(n+2)$ ODEs, which may be written in matrix form as

2.6a $$\begin{aligned} \frac{d}{dt}\underline{\mathbf {x}} = B\underline{\mathbf {x}} + \underline{\mathbf {g}}, \qquad \underline{\mathbf {x}}(0)=\underline{\mathbf {0}}, \end{aligned}$$

where

2.6b $$\begin{aligned} \underline{\mathbf {x}} = \begin{pmatrix} a_{1}(t) \\ a_{2}(t) \\ \vdots \\ \vdots \\ a_{n}(t) \\ a_{b}(t) \\ a_{c}(t) \end{pmatrix}, \;\;\; B = \left( \begin{array}{rrrrrrr} -k &{} &{} &{} &{} &{} &{} \\ k &{} -k &{} &{} &{} &{} &{} \\ &{} k &{} -k &{} &{} &{} &{} \\ &{} &{} \ddots &{} \ddots &{} &{} &{} \\ &{} &{} &{} k &{} -k &{} &{} \\ &{} &{} &{} &{} k &{} -k_{a} &{} \\ &{} &{} &{} &{} &{} k_{a} &{} -k_{e} \end{array} \right) , \;\;\; \underline{\mathbf {g}}=\begin{pmatrix}g_{1}(t) \\ 0 \\ \vdots \\ \vdots \\ 0 \\ 0 \\ 0 \end{pmatrix} \; , \end{aligned}$$

and

2.6c $$\begin{aligned} g_{1}(t) = {\left\{ \begin{array}{ll} FD_{0} \delta (t) &{} \text { for regimen (B1)} \\ g_{B}(t) &{} \text { for regimen (Beq)} \\ g_{BL}(t) &{} \text { for regimen (BeqL)} \end{array}\right. } , \end{aligned}$$

where $\delta$ is the Dirac delta function.

For all cases, the solution to the IVP consists of all state variables as functions of time. The primary state variable of interest is the central circulation drug level $a_{c}(t)$.

## Analytical solutions for equi-dosing regimens

Here we present exact solutions for central compartment drug levels under a variety of equi-dosing regimens. Single-compartment and two-compartment model solutions are included for comparison with transit compartment model (TCM) solutions, and to aid the construction of equi-dosing regimen regions in ‘Results: equi-dosing regimen regions’. The exact TCM solutions represent an improvement on existing approximate solutions [44].

**Table 2:** Exact solutions to pertinent single-compartment and two-compartment models under equi-dosing regimen inputs

Singl-compartment IV equi-bolus dosing (M1,Beq):
$$\begin{array}{ll} a_{c}(t) = \textstyle F D_{0}\sum _{j=1}^{M} H\big (t_{j}) e^{-k_{e}t_{j}}, \end{array}$$ (3.1)
$$\begin{array}{ll} a_{c}^{M}(t_{M}) = \textstyle FD_{0}\left( \frac{1-e^{-Mk_{e}T}}{1-e^{-k_{e}T}}\right) e^{-k_{e} t_{M}}, \;\; \text {for } 0\le t_{M} <T, \end{array}$$ (3.2)
$$\begin{array}{ll} a_{c}^{\infty }(t_{\infty }) = \textstyle \left( \frac{FD_{0}}{1-e^{-k_{e}T}}\right) e^{-k_{e} t_{\infty } }, \;\; \text {for } 0\le t_{\infty } <T. \end{array}$$ (3.3)
Single-compartment IV equi-bolus dosing with loading dose (M1,BeqL), with $a_{c}^{\infty }(t_{\infty })$ given by (3.3):
$$\begin{array}{ll} a_{c}(t) = \textstyle F \left\{ D_{0}\left( \sum _{j=1}^{M} H\big (t_{j}) e^{-k_{e}t_{j}}\right) + (D_{L}-D_{0})e^{-k_{e}t}\right\} , \end{array}$$ (3.4)
$$\begin{array}{ll} a_{c}^{M}(t_{M}) = \textstyle F \left\{ D_{0}\left( \frac{1-e^{-Mk_{e}T}}{1-e^{-k_{e}T}}\right) e^{-k_{e} t_{M}} + (D_{L}-D_{0})e^{-k_{e}(t_{M}+(M-1)T)} \right\} . \end{array}$$ (3.5)
Single-compartment IV equi-infusion dosing (M1,Ieq):
$$\begin{array}{ll} a_{c}(t) = \textstyle \frac{Fk_{in}}{k_{e}} \sum _{j=1}^{M} H(t_{j})(1-e^{-k_{e}t_{j}})- H(t_{j}-t_{f})(1-e^{-k_{e}(t_{j}-t_{f})}), \end{array}$$ (3.6)
$$\begin{array}{ll} a_{c}^{M}(t_{M}) = \textstyle \frac{Fk_{in}}{k_{e}} \left\{ (1-e^{-k_{e}t_{M}}) - H(t_{M}-t_{f})(1-e^{-k_{e}(t_{M}-t_{f})}) + (e^{k_{e}t_{f}}-1) \left( \frac{e^{-Mk_{e}T}-e^{-k_{e}T}}{e^{-k_{e}T}-1}\right) e^{-k_{e}t_{M}} \right\} . \end{array}$$ (3.7)
$$\begin{array}{ll} a_{c}^{\infty }(t_{\infty }) = \textstyle \frac{Fk_{in}}{k_{e}} \left\{ 1-\frac{e^{k_{e}t_{f}}-e^{k_{e}T}}{1-e^{k_{e}T}} e^{-k_{e}t_{\infty }} - H(t_{\infty }-t_{f})(1-e^{-k_{e}(t_{\infty }-t_{f})}) \right\} , \;\; \text {for } 0\le t_{\infty } <T. \end{array}$$ (3.8)
Two-compartment oral equi-bolus dosing (M2,Beq):
$$\begin{array}{ll} a_{c}(t) = \textstyle \frac{k_{a}}{k_{e}-k_{a}} FD_{0}\sum _{j=1}^{M} H\big (t_{j}) \left[ e^{-k_{a}t_{j}} - e^{-k_{e}t_{j}}\right] , \end{array}$$ (3.9)
$$\begin{array}{ll} a_{c}^{M}(t_{M}) = \textstyle \frac{k_{a}}{k_{e}-k_{a}} FD_{0} \left[ \left( \frac{1-e^{-Mk_{a}T}}{1-e^{-k_{a}T}}\right) e^{-k_{a}t_{M}} - \left( \frac{1-e^{-Mk_{e}T}}{1-e^{-k_{e}T}}\right) e^{-k_{e}t_{M}} \right] , \end{array}$$ (3.10)
$$\begin{array}{ll} a_{c}^{\infty }(t_{\infty }) = \textstyle \frac{k_{a}}{k_{e}-k_{a}} FD_{0} \left[ \frac{e^{-k_{a}t_{\infty }}}{1-e^{-k_{a}T}} - \frac{e^{-k_{e}t_{\infty }}}{1-e^{-k_{e}T}} \right] . \end{array}$$ (3.11)
Two-compartment oral equi-bolus dosing with loading dose (M2,BeqL), with $a_{c}^{\infty }(t_{\infty })$ given by (3.11):
$$\begin{array}{ll} a_{c}(t) = \textstyle \frac{k_{a}}{k_{e}-k_{a}} F \left\{ D_{0}\sum _{j=1}^{M} H\big (t_{j}) \left[ e^{-k_{a}t_{j}} - e^{-k_{e}t_{j}}\right] + (D_{L}-D_{0})\left[ e^{-k_{a}t} - e^{-k_{e}t}\right] \right\} , \end{array}$$ (3.12)
$$\begin{array}{ll} a_{c}^{M}(t_{M}) = \textstyle \frac{k_{a}F}{k_{e}-k_{a}} \Big \{ D_{0} \left[ \left( \frac{1-e^{-Mk_{a}T}}{1-e^{-k_{a}T}}\right) e^{-k_{a}t_{M}} - \left( \frac{1-e^{-Mk_{e}T}}{1-e^{-k_{e}T}}\right) e^{-k_{e}t_{M}} \right] \nonumber \\ \textstyle \qquad \qquad \qquad + \quad (D_{L}-D_{0})\left[ e^{-k_{a}(t_{M}+(M-1)T)} - e^{-k_{e}(t_{M}+(M-1)T)}\right] \Big \}. \end{array}$$ (3.13)

Models and dosing inputs are as in Table 1, *H* is the Heaviside function, and $t_{j}$ is given by (3.14)

### Exact solutions for one-compartment and two-compartment models with equi-dosing

In Table 2, we list exact solutions for drug level in the central compartment $a_{c}(t)$ for one-compartment and two-compartment model IVPs formulated in Table 1, under equi-dosing inputs given by Fig. 2 and Eqs. (2.3)–(2.5). These include well-known solutions (e.g., [6, 19, 45]); for comparison with the TCM solution, their derivations may be found in detail in Appendix 1. Throughout, *H* is the Heaviside function, and

3.14 $$\begin{aligned} t_{j}=t-(j-1)T = \text { time since } j{\text {th}} \text { dose}. \end{aligned}$$

Solutions may be written compactly without summation notation by considering, for example, the central compartment drug level after the *M*th dose, $a_{c}^{M}(t_{M})$. The steady-state (*T*-periodic) drug level function is denoted $a_{c}^{\infty }(t_{\infty })$, where $t_{\infty }$ is the time since the start of the dosing interval.

### Exact solution for transit compartment model with equi-bolus dosing

More generally, the transit compartment model (Mt) comprises a multi-compartment oral absorption process, and the ODEs may be written in matrix form as in (2.6). We consider equi-bolus dosing, i.e. forcing input (Beq), so that $g_{1}(t)=g_{B}(t)$. The exact solution may be written using the matrix exponential [9, 27], or by using Laplace Transforms (Appendix 1.4). For the transit compartments, we find that

3.15 $$\begin{aligned} a_{i}(t) \;\; = \;\; \frac{F D_{0}k^{i-1}}{(i-1)!} \sum _{j=1}^{M} H(t_{j}) t_{j}^{i-1} e^{-kt_{j}}, \qquad i=1, \ldots , n, \end{aligned}$$

where $t_{j}=t-(j-1)T$. We note that this result is equivalent to the multi-dose analytical result of Shen *et al* [46] which was derived via superposition arguments, to be used as an input to their central compartment module, and also to the Savic single-dose solution [44] if $M=1$. We now extend our calculations to establish solutions for the absorption and central compartment drug levels, which in effect gives a general, analytical multi-dose solution to the Savic problem [44]. The solution for the absorption compartment (see Appendix 1.4) is

3.16 $$\begin{aligned} a_{b}(t) = \frac{F D_0}{(n-1)!} \left( \frac{k}{k-k_{a}}\right) ^{n} \sum _{j=1}^{M} H(t_{j}) e^{-k_{a}t_{j}} \, \gamma \big (n,(k-k_{a})t_{j}\big ), \end{aligned}$$

where $\gamma$ is the *lower incomplete gamma function*, defined by (for positive integer *n*, see [3])

3.17 $$\begin{aligned} \gamma (n,t) \;\; = \;\; \int _{0}^{t} x^{n-1}e^{-x} \; dx \; \;\; = \;\; (n-1)! \left( 1-e^{-t}\sum _{p=0}^{n-1} \frac{t^{p}}{p!} \right) . \end{aligned}$$

Finally, the drug level in the central compartment (the primary output of interest), $a_{c}(t)$, is given by (Appendix 1.4):

3.18 $$\begin{aligned}&a_{c}(t) \;\; = \;\; \frac{FD_{0}k^{n}k_{a}}{(n-1)!(k_{e}-k_{a})} \sum _{j=1}^{M} H\left( t_{j}\right) \, \left\{ \frac{e^{-k_{a}t_{j}}}{(k-k_{a})^{n}} \gamma \big (n,(k-k_{a})t_{j}\big ) \;\right. \nonumber \\&\left. \quad - \; \frac{e^{-k_{e}t_{j}}}{(k-k_{e})^{n}} \gamma \big (n,(k-k_{e})t_{j}\big ) \right\} . \end{aligned}$$

We next seek the steady-state solutions, as we have for the one- and two-compartment problems.

#### Steady-state behaviour

The derivation of the steady-state solution is more involved than for the earlier models (see Appendix 1.4.1). For the transit compartments, we find that

3.19 $$\begin{aligned} a_{i}^{\infty }(t_{\infty }) = \left( \sum _{p=0}^{i-1} \frac{a_{i-p}^{\infty }(0)}{p!} (kt_{\infty })^{p} \right) e^{-k t_{\infty }}, \qquad \text {for } i=1, \ldots , n. \end{aligned}$$

The coefficients $a_{i}(0)$ (the steady-state dosing interval initial values) may be found using

3.20 $$\begin{aligned} a_{1}^{\infty }(0) = \frac{FD_{0}}{1-e^{-\phi }}. \end{aligned}$$

together with the recurrence relation (for $i=2, \ldots , n$)

3.21 $$\begin{aligned} a_{i}(0)= & {} \beta \times \left( \frac{a_{i-1}^{\infty }(0)}{1!}\, \phi + \frac{a_{i-2}^{\infty }(0)}{2!}\, \phi ^{2} + \frac{a_{i-3}^{\infty }(0)}{3!}\, \phi ^{3}\right. \nonumber \\&\left. + \cdots + \frac{a_{1}^{\infty }(0)}{(i-1)!}\, \phi ^{i-1}\right) \;\; = \;\; \beta \sum _{p=1}^{i-1} \frac{a_{i-p}^{\infty }(0)}{p!}\phi ^{p}, \end{aligned}$$

where

3.22 $$\begin{aligned} \phi =kT, \qquad \text {and} \qquad \beta =\frac{e^{-\phi }}{1-e^{-\phi }}. \end{aligned}$$

Computationally, we may use this recurrence relation. Further, a closed form expression for $a_{i}^{\infty }(0)$ is found (see Appendix 1.4.1):

3.23 $$\begin{aligned}&a_{i}^{\infty }(0) = \frac{FD_{0}}{1-e^{-\phi }}\, \frac{\phi ^{i-1}}{(i-1)!} \sum _{p=0}^{i-1} p! S(i-1,p)\beta ^{p},\nonumber \\&\quad \text {for } i=1, \ldots , n, \end{aligned}$$

where *S* is the Stirling number of the second kind [40], given by

3.24 $$\begin{aligned} S(n,q)=\frac{1}{q!}\sum _{p=0}^{q} (-1)^{p}\, \begin{pmatrix} q \\ p \end{pmatrix} (q-p)^{n}, \qquad \text {where} \quad \begin{pmatrix} q \\ p \end{pmatrix}=\frac{q!}{p!(q-p)!} \text {~~ is the binomial coefficient}, \end{aligned}$$

and taking $S(0,0)=1$. For the absorption compartment, we find

3.25 $$\begin{aligned} a_{b}^{\infty }(t_{\infty })= & {} \left[ a_{b}^{\infty }(0) + \sum _{p=1}^{n} \frac{a_{p}^{\infty }(0)}{(n-p)!} \left( \frac{k}{k-k_{a}}\right) ^{n-p+1} \,\right. \nonumber \\&\left. \gamma (n-p+1,(k-k_{a})t_{\infty }) \right] e^{-k_{a}t_{\infty }}, \end{aligned}$$

where

3.26 $$\begin{aligned}&a_{b}^{\infty }(0) = \beta _{a}\sum _{p=1}^{n} \frac{a_{p}^{\infty }(0)}{(n-p)!} \left( \frac{k}{k-k_{a}}\right) ^{n-p+1} \,\nonumber \\&\quad \gamma (n-p+1,(k-k_{a})T) , \end{aligned}$$

and

3.27 $$\begin{aligned} \beta _{a} = \frac{e^{-k_{a}T}}{1-e^{-k_{a}T}}. \end{aligned}$$

Finally, and ultimately, the steady-state profile in the central compartment is given by

3.28 $$\begin{aligned} \begin{aligned} a_{c}^{\infty }(t_{\infty })&= a_{c}^{\infty }(0)e^{-k_{e}t_{\infty }} + \frac{k_{a}}{k_{a}-k_{e}}\\&\quad \times \Bigg \{ a_{b}^{\infty }(0)(e^{-k_{e}t_{\infty }}-e^{-k_{a}t_{\infty }}) \; + \; \Bigg . \\&\; \sum _{p=1}^{n} \frac{a_{p}^{\infty }(0)}{(n-p)!} \left[ \left( \frac{k}{k-k_{e}}\right) ^{n-p+1} \, e^{-k_{e}t_{\infty }} \,\right. \\&\left. \quad \gamma (n-p+1,(k-k_{e})t_{\infty }) \; - \; \right. \\&\quad \left( \frac{k}{k-k_{a}}\right) ^{n-p+1} \, e^{-k_{a} t_{\infty }} \,\left. \gamma (n-p+1,(k-k_{a})t_{\infty }) \right] \Bigg . \Bigg \} , \end{aligned} \end{aligned}$$

where

3.29 $$\begin{aligned} \begin{aligned} a_{c}^{\infty }(0)&= \beta _{c} \times \Bigg \{ a_{b}^{\infty }(0)(e^{-k_{e}T}-e^{-k_{a}T}) \; + \; \Bigg . \\&\; \sum _{p=1}^{n} \frac{a_{p}^{\infty }(0)}{(n-p)!} \left[ \left( \frac{k}{k-k_{e}}\right) ^{n-p+1} \, e^{-k_{e}T} \, \gamma (n-p+1,(k-k_{e})T) \; - \; \right. \\&\qquad \qquad \qquad \left. \left( \frac{k}{k-k_{a}}\right) ^{n-p+1} \, e^{-k_{a} T} \, \gamma (n-p+1,(k-k_{a})T) \right] \Bigg . \Bigg \} , \end{aligned} \end{aligned}$$

and

3.30 $$\begin{aligned} \beta _{c} = \frac{k_{a}}{(k_{a}-k_{e})(1-e^{-k_{e}T})}. \end{aligned}$$

We now have in place new analytical solutions for a general *M*-equi-dose input to an *n*-transit compartment model with absorption and central compartments. These solutions may be used to predict drug level dynamics exactly, rather than approximately (see (4.5)). Further, the steady-state solutions may be used to guide safe and effective dosing regimen design, given a specified therapeutic range.

#### Computational evaluation of the lower incomplete gamma function

In order to use the analytical results of the previous subsection, a computational method for evaluating the lower incomplete gamma function is required. The definition itself (3.17) immediately suggests several approaches for a given *n*, including numerical evaluation of the integral for given *t*, computation of the truncated exponential sum, and using symbolic computation to derive an exact expression for the integral, then evaluating at given *t*. In fact, more efficient methods for evaluating this function have received attention in the mathematical literature, with many involving series and continued fraction expansions [1, 2, 17, 41, 50]. In MATLAB, the built-in function gammainc may be used [35], and is our preferred evaluation method due to its accuracy and run time (see Appendix 1.4.2). In software where such a function is not avaialble, the following relationship between the lower incomplete gamma function $\gamma$ and the cumulative distribution function $F_{\varGamma }$ for the gamma distribution may be used ( [35]):

3.31 $$\begin{aligned} \gamma (n,t) = \varGamma (n)F_{\varGamma }(t;n,1). \end{aligned}$$

Here, *n* is taken as the shape parameter of the distribution, and the scale parameter is unity. Furthermore, the log-gamma function is a built-in function in many software packages, and the exponentiated log-gamma function is often used in situations where numerical difficulties may arise in evaluating the gamma function directly [35]. Hence, a practical approach for evaluating $\gamma (n,t)$ is to use built-in functions to evaluate

3.32 $$\begin{aligned} \gamma (n,t) = e^{\ln \varGamma (n)} F_{\varGamma }(t;n,1) = \exp (\ln \varGamma (n)) F_{\varGamma }(t;n,1). \end{aligned}$$

The log-gamma function is available in PK analysis packages and languages including NONMEM [4], MLXTRAN/MONOLIX [31], PharmML [48], and also in MATLAB [35] and Excel [37]. Each of these packages also has the exponential function and cumulative gamma distribution *F* available.

## Results: equi-dosing time courses

Here, we present simulated time courses of drug levels, using the analytical solutions given in ‘Analytical solutions for equi-dosing regimens’. In all cases, we have computed using MATLAB [35].

### IV equi-bolus dosing—one-compartment model

**Fig. 3:**
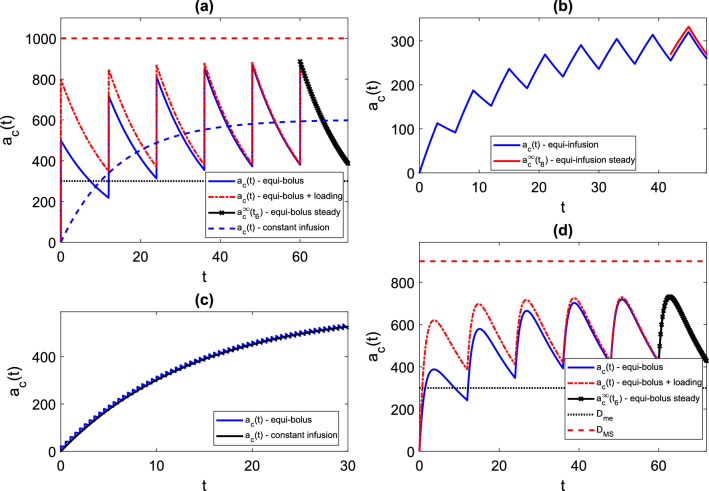
Drug level time courses. Throughout, we take $F=1$. Where shown, $D_{me}$ and $D_{MS}$ represent hypothetical minimum effective and maximum safe levels respectively, giving the therapeutic range $[D_{me},D_{MS}]$. Where shown, the steady-state profile overlays the final dosing interval for comparison. **a** IV equi-bolus dosing (M1, Beq), with and without loading dose. $D_{0}$=500 mg, *T* = 12 h, $k_{e}=0.0692$ h$^{-1}$ (taken from [10]). Loading dose (M1,BeqL) has $D_{L}=800$ mg. Continuous infusion at a rate $k_{in}=\frac{D_{0}}{T}=41.67$ h$^{-1}$ is also shown. **b** IV equi-infusion dosing (M1,Ieq) with $k_{in}=41.67$ h$^{-1}$, $t_{f}=3$ h, $T=6$ h, $k_{e}$=0.0692 h$^{-1}$. **c** IV equi-bolus dosing (M1, Beq) with $D_{0}$=20.83 mg, *T*=0.5h, $k_{e}=0.0692$h $^{-1}$, together with continuous infusion with $k_{in}=\frac{D_{0}}{T}=41.67$ h$^{-1}$. (d) Oral equi-bolus dosing (M2,Beq) with $D_{0}$ = 500 mg, *T*=12h, $k_{e}=0.0692$ h$^{-1}$, $k_{a}=0.7$ h$^{-1}$. Loading dose (M2,BeqL) has $D_{L}=800$ mg

In Fig. 3a, we plot a typical drug level time course for the IV equi-bolus dosing problem (M1,Beq), which has solution given by (3.2), and steady-state profile given by (3.3). The characteristic features of the time courses include jump discontinuities at $t=jT$, exponential decay over each dosing interval, and approach to a periodic steady-state [45]. Clearly, acceptable dosing regimens would only exist for certain $(T,D_{0})$ choices. Also shown is the continuous infusion profile given by

4.1 $$\begin{aligned} a_{c}(t)=\frac{Fk_{in}}{k_{e}}(1-e^{-k_{e}t}), \end{aligned}$$

taking $k_{in}=\frac{D_{0}}{T}$ (see (A.16)). The dosing interval average drug level approaches the corresponding infusion steady-state level, which is apparent from the graph, and from calculating $\overline{a_{c}^{\infty }}_{[0,T]}=\frac{F\times \frac{D_{0}}{T}}{k_{e}}$ from (3.3) and comparing with the steady-state of (4.1). Finally, we note that the administration of a loading dose at time $t=0$ may give a treatment that is immediately and always therapeutic, and drug levels close to the steady-state.

### IV equi-infusion dosing

In Fig. 3b, we plot a typical drug level time course for the IV equi-infusion dosing problem (M1,Ieq), which has solution given by (3.6), and steady-state profile given by (3.8). The characteristic features of the time courses include derivative discontinuities (but continuous drug levels) at $t=jT$ and $t=jT+t_{f}$, exponentially decaying rise followed by exponential decay over each dosing interval, and approach to a periodic steady-state [45]. Again, acceptable dosing regimens would only exist for certain $(T,D_{0})$ choices.

### Single continuous infusion as limit of IV equi-bolus dosing

In Fig. 3c, we plot a drug level time course for IV continuous infusion (4.1) for infusion rate $k_{in}$, together with an equi-bolus dosing (M1,Beq) solution (3.2) for which the dosing rate is $\frac{D_{0}}{T}=k_{in}$ with very short dosing interval *T*. It is apparent, and intuitively known, that continuous infusion represents a limit of a corresponding equi-bolus regimen for high dosing frequency. In Appendix 2.1 we offer a proof of this result using l’Hopital’s rule.

### Oral bolus equi-dosing—two-compartment model

In Fig. 3d, we plot a typical drug level time course for the oral equi-bolus dosing problem (M2,Beq), which has solution given by (3.10), and steady-state profile given by (3.11). The characteristic features of the time courses include derivative discontinuities at $t=jT$, a two-phase profile (absorption then elimination) over each dosing interval, and approach to a periodic steady-state [45]. We note that the administration of a loading dose at time $t=0$ may give a treatment that reaches therapeutic level earlier, but for which there is still some nonzero waiting time before the therapeutic level is reached. It is clearly possible to give a loading dose which ensures that therapeutic drug level is both reached within the first dosing interval and is maintained thereafter.

The $a_{c}(t)$ time course approaches a *T*-periodic steady-state profile, which will be non-monotonic for all regimens (even those for which the time course is monotonic for the early dosing intervals), exhibiting both absorption and elimination phases. Expressions for the peak drug level and the peak timing are given in Appendix 2.2. Maximum and minimum drug levels for a number of models will be used in ‘Results: equi-dosing regimen regions’ to construct *equi-dosing regimen regions*, which give a summary guide for regimen design.

### Transit compartments—smoothed delays, lag time and data fitting (single-dose)

**Fig. 4:**
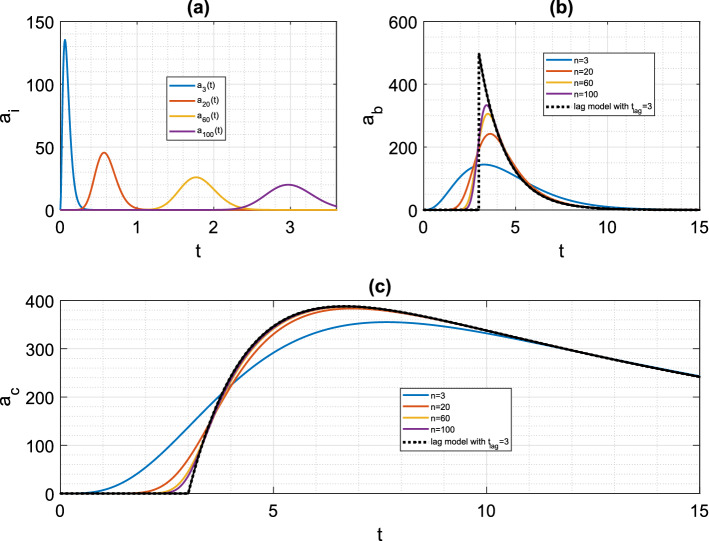
Drug level time courses for transit compartment model with a single dose (Mt,B1). Throughout, we take $$F=1$$, $$D_{0}$$ = 500 mg, $$k_{e}$$=0.0692 h$$^{-1}$$ $$k_{a}$$=0.7 h$$^{-1}$$ (for hypothetical drug described in [10]). Here, $$t_{lag}=MTT=3$$h, and $$k=\frac{MTT}{n}$$. **a** Drug level $$a_{i}(t)$$ for compartments $$i=3, 20, 60, 100$$ of a cascade with $$n=100$$ transit compartments. **b** Absorption compartment level $$a_{b}(t)$$ for cascades with $$n=3, 20, 60, 100 $$ transit compartments, with equivalent time-lag profile for pure delay to absorption compartment. **c** Central compartment level $$a_{c}(t)$$ for cascades with $$n=3, 20, 60, 100 $$ transit compartments, with equivalent time-lag profile for pure delay to absorption compartment

In Fig. 4, we demonstrate the delaying effect of a train of transit compartments for a single dose regimen. It is clear (Fig. 4a) that a delta-function-like bolus dose input to the first transit compartment effects a “spread-out bolus” dose in later transit compartments. Eventually a spread-out bolus profile is seen for the final transit compartment, which becomes the input to the absorption compartment, centered around $t=MTT=\frac{n}{k}$. As in [44], we consider the transit compartment cascade delaying the appearance of bolus dose in the absorption compartment of a standard two-compartment oral dosing model. We see that (Fig. 4b, c), for a fixed time lag $t_{lag}=MTT$ taking $k=\frac{MTT}{n}$, the pure delay (time-lag) profiles (see [45])

4.2 $$\begin{aligned} a_{b}^{lag}(t)={\left\{ \begin{array}{ll} 0 &{} 0\le t \le t_{lag} \\ FD_{0}e^{-k_{a}(t-t_{lag})} &{} t>t_{lag} \end{array}\right. }, \end{aligned}$$

and

**Fig. 5:**
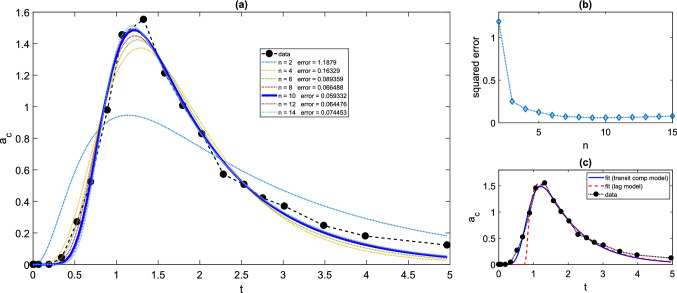
Drug level time courses for transit compartment model with a single dose (Mt,B1), with fitting to experimental data for drug glibenclamide, taken from [44], using WebPlotDigitizer [42], in response to a 3.5 mg dose. Original data converted from concentration to drug level using volume of distribution of 3.79l [44]. Central compartment drug level (mg) versus time (h) is shown. **a** Time courses fitting model (Mt,B1) to data for varying number of transit compartments *n*. **b** Sum of squared errors between data and best-fit simulation for $2\le n \le 15$. **c** Best-fit transit compartment and lag-time models, together with time course data. For (Mt,B1) model, $n=10$ gives best fit, with fitted parameters $k=12.76$ h$^{-1}$, $k_{a}=9.11$ h$^{-1}$, $k_{e}=0.96\,$h$^{-1}$, $F=0.69$, and sum of squared errors 0.059. For time-lag model, fitted parameters are $k_{a}=5.67\,$h$^{-1}$, $k_{e}=0.92\,$h$^{-1}$, $t_{lag}=0.78$h and $F=0.63$, and sum of squared errors 0.38

4.3 $$\begin{aligned} a_{c}^{lag}(t)={\left\{ \begin{array}{ll} 0 &{} 0\le t \le t_{lag} \\ \frac{k_{a}}{k_{e}-k_{a}}FD_{0}\left( e^{-k_{a}(t-t_{lag})} - e^{-k_{e}(t-t_{lag})} \right) &{} t>t_{lag} \end{array}\right. }, \end{aligned}$$

are approached by the equivalent transit compartment approximations for increasing *n*. Such “smoothed delay” profiles may well capture experimental data better than no-delay or pure-delay models [44]. Indeed, we find a better fit to published data for a single dose of the drug glibenclamide [44] using a transit compartment model than using a pure time-lag model (Fig. 5). For the least-squares data fitting shown in Fig. 5, we use the optimisation function fminsearch in MATLAB [35], with the objective function being the sum of squares between data and simulation at the data points. For each fixed *n* in turn, and for the time-lag model (for which $t_{lag}$ is one of the fitted parameters), the optimisation routine is run for 1000 iterations.

#### Parameter identifiability

We note that applying optimisation routines to estimate PK parameters for $n=1$ (a single transit compartment) should be with caution, since in this case, $k_{e}$ is identifiable but *k* and $k_{a}$ are unidentifiable. This is readily seen by considering a single bolus dose to the transit compartment for cases (i) $k=k_{1}$ and $k_{a}=k_{2}$, and (ii) $k=k_{2}$ and $k_{a}=k_{1}$, for rate constants $k_{1}$ and $k_{2}$. In both cases, the inflow rate to the central compartment $k_{a} a_{b}(t)$ is found from (3.15) to (3.16) to be

4.4 $$\begin{aligned} k_{a} a_{b}(t) = FD_{0} \frac{k_{1}k_{2}}{k_{2}-k_{1}}\left[ e^{-k_{1}t}-e^{-k_{2}t}\right] . \end{aligned}$$

The central compartment drug level will be identical for both cases, hence $k_{a}$ and *k* are not uniquely identifiable from the single output $a_{c}(t)$. This is a manifestation of the so-called flip-flop phenomenon for two-compartment kinetics [45]. The parameter $k_{e}$ is identifiable. An example computation is shown in Appendix 2.3. For $n>1$ this phenomenon is avoided, and all parameters are uniquely identifiable (see Appendix 2.3).

### Transit compartments—exact versus approximate solutions

The original transit compartment schematic presented in [44] has been key to our analysis. A significant advance in our work is the development of an analytical solution which solves the problem exactly. The original single bolus dose analysis [44] uses exact solutions for each transit compartment, but employs the Stirling approximation for the factorial, to solve the following system for the final two compartments.

4.5a $$\begin{aligned} \frac{da_{b}}{dt}&= \frac{FD_{0}k^{n}}{\sqrt{2\pi } (n-1)^{n-\frac{1}{2}}e^{-(n-1)}} t^{n-1}e^{-kt}, \end{aligned}$$

4.5b $$\begin{aligned} \frac{da_{c}}{dt}&= k_{a}a_{b} -k_{e} a_{c}, \end{aligned}$$

4.5c $$\begin{aligned} a_{b}(0)&=a_{c}(0)=0. \end{aligned}$$

**Fig. 6:**
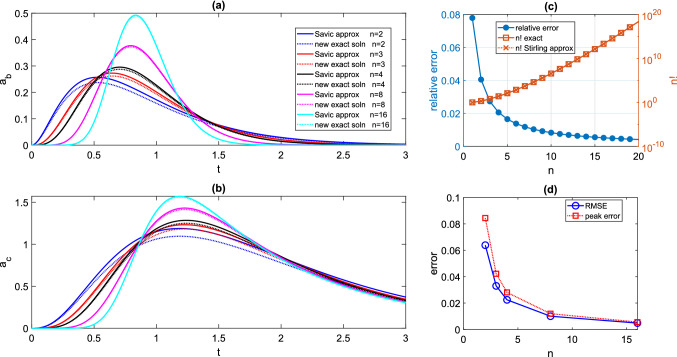
Difference between exact and approximate solutions for transit compartment model with single bolus dose. Throughout, we take $k_{a}=9.11\,$h$^{-1}$, $k_{e}=0.96\,$h$^{-1}$, $F=0.69$, $D_{0}=3.5\,mg$, and $MTT=0.78$h, and for each *n*, we take $k=\frac{n}{MTT}$. **a** Absorption compartment drug level - exact (3.16) versus approximate (4.5) solutions for varying *n*. **b** Central compartment drug level—exact (3.18) versus approximate (4.5) solutions for varying *n*. **c** Relative error made using Stirling approximation of *n*!. (d) Root mean squared error (RMSE) and relative error between exact and approximate solution peak values for $a_{c}(t)$ shown in panel (b)

A comparison between a typical numerical solution of the approximate model (4.5) and corresponding exact solutions given by (3.16)–(3.18) for a single bolus dose is given in Fig. 6. It is clear that the new exact solutions provide a significant improvement in accuracy over the approximate solutions, particularly for $n\le 4$, for which the relative error in the peak $a_{c}$ value is between 3% and 8.4%.

### Transit compartments—equi-dosing

**Fig. 7:**
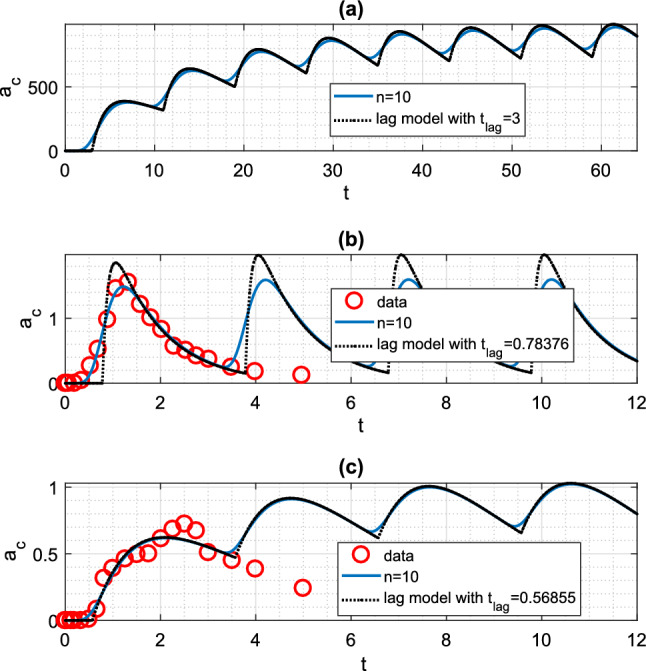
Equi-dosing drug level $a_{c}(t)$ time courses for transit compartment model (Mt,Beq). **a** Simulated time course (Mt,B1) for $n=10$ transit compartments with $MTT=3$h. Here, $F=1$, $k_{e}$=0.0692 h$^{-1}$ $k_{a}$=0.7 h$^{-1}$ (for hypothetical drug described in [10]). Equi-dosing regimen has $D_{0}$ = 500 mg, *T*=8h. Also shown is equivalent pure time lag result. **b** Simulated time course for parameters fitted to Savic single dose data [44] for glibenclamide and (Mt,B1) model, as in Fig. 5. Equi-dosing regimen has $D_{0}$ = 3.5 mg, *T*=3h. Also shown are equivalent pure time lag result, and data points used for fitting. **c** Simulated time course for parameters fitted to a Savic single dose time course (for a different individual) digitised from [44] for glibenclamide and (Mt,B1) model. Equi-dosing regimen has $D_{0}$ = 3.5 mg, *T*=3h. Also shown are equivalent pure time lag result, and data points used for fitting. For (Mt,B1) model, $n=10$ gives best fit, with fitted parameters $k=17.59\,$h$^{-1}$, $k_{a}=0.87\,$h$^{-1}$, $k_{e}=0.48\,$h$^{-1}$, $F=0.37$

While the published data and modelling in [44] focus on a single dose, we naturally wish to use such models for simulating time courses under multi-dosing regimens. The local maxima and minima in a multi-dosing timecourse prediction as the system approaches a periodic steady-state must be considered in regimen design [12]. In Fig. 7, we show predicted equi-dosing drug levels together with data used for fitting to a single dose, as in [12]. The approach to a periodic steady-state is clear in each case. For each timecourse, we note that the transit compartment profile is bounded by the pure time delay profile as we approach steady-state. So using a pure time lag model to fit single dose data and predict multi-dosing dynamics may overestimate the level of fluctuation, which is an important characteristic considered in regimen design.

In Appendix 2.4, we present further time course simulations illustrating the effect of the transit rate constant on the smoothed delay through the system.

## Results: equi-dosing regimen regions

It is common to consider potential therapeutic protocols which will give steady-state drug levels within a prescribed therapeutic range by simulation with multiple dosing regimens and observing whether the steady-state falls within that therapeutic range [12, 25, 43, 45]. One can approach dosing regimen design iteratively, simulating in this manner and adjusting base parameters until a theoretically safe and therapeutic regimen is found [25]. Here we present a novel and alternative analysis which will capture the *constraints* imposed on the dosing regimen parameters by the therapeutic range, which is given by

5.1 $$\begin{aligned} D_{me}=\,& {} \text {minimum effective level and} \qquad \\ D_{MS}\nonumber=\,& {} \text {maximum safe level}. \end{aligned}$$

We propose that equi-dosing regimen regions (EDRRs), which are regions of the parameter space giving acceptable regimens, may be used to summarise steady-state constraints and guide regimen design from the outset of any investigation.

### Two-parameter dosing regimens

#### Equi-dosing regimen region for IV equi-bolus dosing

For model (M1) with forcing (Beq), we seek constraints on the two equi-dosing regimen parameters $D_{0}$ and *T* such that the steady-state time course given by (3.3) has

5.2 $$\begin{aligned} D_{me}<a_{c}^{\infty }(t_{\infty })<D_{MS}. \end{aligned}$$

Now $a_{c}^{\infty }(0)=\frac{FD_{0}}{1-e^{-k_{e}T}}$ and $a_{c}^{\infty }(T^{-})=\frac{FD_{0}e^{-k_{e}T}}{1-e^{-k_{e}T}}$, with $a_{c}^{\infty }(t_{\infty })$ decreasing, so we require that

$$\begin{aligned} D_{me}<\frac{FD_{0}e^{-k_{e}T}}{1-e^{-k_{e}T}} \qquad \text {and} \qquad \frac{FD_{0}}{1-e^{-k_{e}T}} < D_{MS}, \end{aligned}$$

so that the region of $(T,D_{0})$-space for acceptable dosing regimens is that corresponding to the inequality constraints

5.3 $$\begin{aligned} \frac{D_{me}}{F}\left( e^{k_{e}T}-1\right) \; \;< D_{0} \;\; < \;\; \frac{D_{MS}}{F}\left( 1-e^{-k_{e}T}\right) . \end{aligned}$$

The upper bounds on *T* and $D_{0}$ for the safe and effective dosing region are given by

5.4 $$\begin{aligned} T=\frac{1}{k_{e}}\log \left( \frac{D_{MS}}{D_{me}}\right) , \qquad D_{0}=\frac{1}{F}(D_{MS}-D_{me}). \end{aligned}$$

**Fig. 8:**
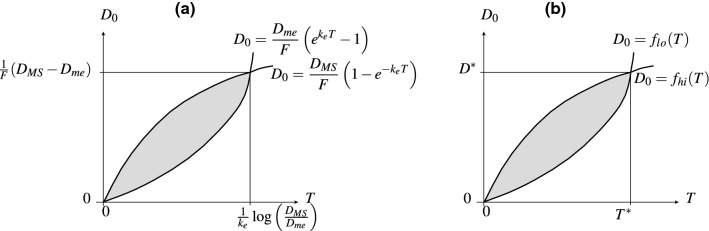
The two-dimensional equi-dosing regimen regions (EDRRs, the shaded, petal-shaped regions) for **a** IV equi-bolus dosing, and **b** oral equi-bolus dosing. Functions $f_{lo}$ and $f_{hi}$ are given in (5.6)

**Fig. 9:**
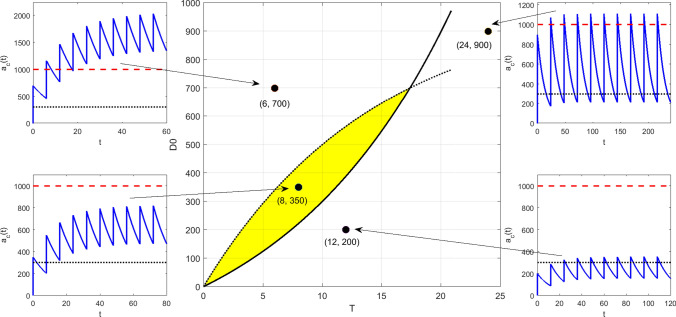
Equi-dosing regimen region (EDRR) for IV dosing with $F=1$, $k_{e}=0.0692h^{-1}$, and hypothetical minimum effective and maximum safe drug levels $D_{me}=300$ mg and $D_{MS}=1000$ mg. Sample time courses $a_{c}(t)$ for four $(T,D_{0})$ regimens are shown, illustrating four different possibilities for the steady-state drug level: (i) unsafe (toxic, overshooting therapeutic range), (ii) acceptable (safe and effective, entirely within therapeutic range), (iii) both overshooting and undershooting therapeutic range, (iv) ineffective (undershooting therapeutic range)

We see in Fig. 8a that the acceptable EDRR for equi-bolus dosing is given by a petal-shaped region. The two curves divide the $(T,D_{0})$ parameter space into four regions; the three regions other than the EDRR correspond to steady-state drug levels which are unsafe, sub-therapeutic, or both unsafe and sub-therapeutic over subintervals of their periodic timecourses. Illustrative drug level time courses are shown in Fig. 9.

Clearly the EDRR could be used at the outset of any investigation to guide regimen design, prior to any time course simulation or experiment.

#### Equi-dosing regimen region for oral equi-bolus dosing

For model (M2) with forcing (Beq), we consider the steady-state solution (3.11). It is straightforward to show (Appendix 2.2) that the minimum and maximum levels $a_{c,\text {min}}^{\infty }$, $a_{c,\text {max}}^{\infty }$, and peak time $t^{*}_{\infty }$ are given by

5.5a $$\begin{aligned} a_{c,\text {min}}^{\infty }&= a_{c}^{\infty }(0) \;\; = \;\; \frac{k_{a}}{k_{e}-k_{a}} FD_{0} \; \; \left\{ \, \frac{1}{1-e^{-k_{a}T}} \; - \; \frac{1}{1-e^{-k_{e}T}} \, \right\} \; , \end{aligned}$$

5.5b $$\begin{aligned} t^{*}_{\infty }&= \frac{1}{k_{a}-k_{e}} \; \log \left( \, \frac{k_{a}}{k_{e}} \, \frac{1-e^{-k_{e}T}}{1-e^{-k_{a}T}}\, \right) \; , \end{aligned}$$

5.5c $$\begin{aligned} a_{c,\text {max}}^{\infty }&= a_{c}^{\infty }\left( t^{*}_{\infty }\right) \;\; = \;\; FD_{0} \left\{ \left( \, \frac{k_{e}}{k_{a}}\right) ^{k_{e}} \; \frac{ \left( 1-e^{-k_{a}T} \right) ^{k_{e}} }{ \left( 1-e^{-k_{e}T} \right) ^{k_{a}} } \, \right\} ^{\frac{1}{k_{a}-k_{e}}} \; . \end{aligned}$$

Again requiring that $D_{me}<a_{c}^{\infty }(t_{\infty })<D_{MS}$, we find that the region of $(T,D_{0})$-space for acceptable dosing regimens is that corresponding to the inequality constraints

5.6 $$\begin{aligned}&\underbrace{\frac{D_{me}}{F} \, \frac{k_{a}-k_{e}}{k_{a}} \left\{ \frac{(1-e^{-k_{e}T})(1-e^{-k_{a}T})}{e^{-k_{e}T}-e^{-k_{a}T}} \right\} }_{f_{lo}(T)} \;\nonumber \\&\quad< \;\; D_{0} \;\; < \; \underbrace{\frac{D_{MS}}{F} \, \left\{ \left( \, \frac{k_{e}}{k_{a}}\right) ^{k_{e}} \; \frac{ \left( 1-e^{-k_{a}T} \right) ^{k_{e}} }{ \left( 1-e^{-k_{e}T} \right) ^{k_{a}} } \, \right\} ^{\frac{1}{k_{e}-k_{a}}}}_{f_{hi}(T)} \; . \end{aligned}$$

**Fig. 10:**
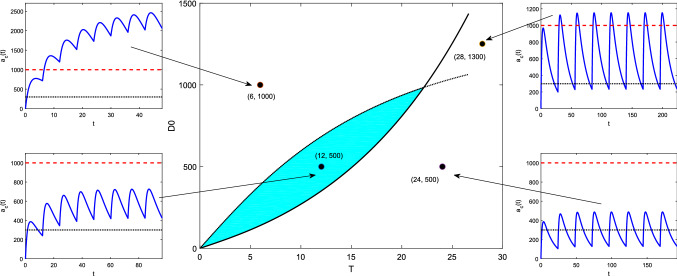
Equi-dosing regimen region (EDRR) for oral dosing with $F=1$, $k_{e}$=0.0692 h$^{-1}$, $k_{a}$=0.7 h$^{-1}$, and hypothetical minimum effective and maximum safe drug levels $D_{me}=300$ mg and $D_{MS}=1000$ mg. Sample time courses $a_{c}(t)$ for four $(T,D_{0})$ regimens are shown, illustrating four different possibilities for the steady-state drug level: (i) unsafe (toxic, overshooting therapeutic range), (ii) acceptable (safe and effective, entirely within therapeutic range), (iii) both overshooting and undershooting therapeutic range, (iv) ineffective (undershooting therapeutic range)

We see in Fig. 8b that the acceptable EDRR for oral equi-bolus dosing is given by another petal-shaped region. The crossover values $D^{*}$ and $T^{*}$ are not easily found analytically as in (5.4) this time; if values are required for these, they may be found numerically. Again the parameter space is divided into four regions which correspond to different safety and effectiveness combinations. A computed EDRR with illustrative drug level time courses is shown in Fig. 10.

#### Equi-dosing regimen region for oral equi-bolus dosing with *n* transit compartments

For the transit compartment model (Mt) with forcing (Beq), we consider the steady-state solution (3.28). Locating extrema in the time course is no longer viable analytically; simple inequalities such as in (5.3) and (5.6) cannot be found. Instead, to construct the EDRR, we discretise the $(T,D_{0})$-space, and compare numerically-found maxima and minima of $a_{c}(t)$ with $D_{MS}$ and $D_{me}$ respectively. The numerical method for constructing transit compartment EDRRs is summarised algorithmically and compared with the analytical approach for the simpler models in Table 3.

**Table 3:** Summary algorithms for constructing two-parameter $(T,D_{0})$ equi-dosing regimen regions

Analytical approach (one- and two-compartment models)	Numerical approach for TCM (construct $f_{lo}(T)$ and $f_{hi}(T)$ numerically)
Find expression for max and min values of $a_{c}^{\infty }$, namely $a_{c,\text {max}}^{\infty }$ and $a_{c,\text {min}}^{\infty }$, in terms of dosing regimen parameters $D_{0}$ and *T*, as in (5.2) and (5.5) Set therapeutic range constraints $D_{me}<a_{c,\text {min}}^{\infty }$ and $a_{c,\text {max}}^{\infty }<D_{MS}$ Rearrange constraints into form $f_{lo}(T)<D_{0}<f_{hi}(T)$, as in (5.3) and (5.6) Plot curves $D_{0}=f_{lo}(T)$ and $D_{0}=f_{hi}(T)$. Region bounded by these two curves is the EDRR	Discretise the $(T,D_{0})$ parameter space, i.e. lay down a grid of points $(T_{i},D_{0,j})$ For each $T_{i}$: For each $D_{0,j}$: Use expression for $a_{c}^{\infty }(t^{\infty })$ (3.28) to compute $a_{c,\text {max}}^{\infty }$ and $a_{c,\text {min}}^{\infty }$ numerically End $f_{lo}(T_{i}) = D_{0,j}$ such that $|a_{c,\text {min}}^{\infty }-D_{me}|$ is minimised $f_{hi}(T_{i}) = D_{0,j}$ such that $|a_{c,\text {max}}^{\infty }-D_{MS}|$ is minimised End Plot curves $D_{0}=f_{lo}(T)$ and $D_{0}=f_{hi}(T)$. Region bounded by these two curves is the EDRR

**Fig. 11:**
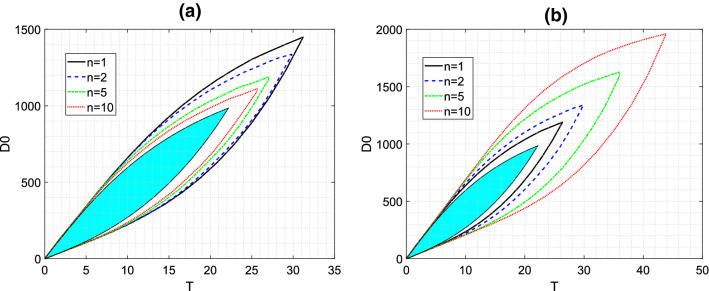
Equi-dosing regimen region (EDRR) for transit compartment model with $F=1$, $k_{e}$=0.0692 h$^{-1}$, $k_{a}$=0.7 h$^{-1}$, and hypothetical minimum effective and maximum safe drug levels $D_{me}=300$ mg and $D_{MS}=1000$ mg. In both plots, the solid light blue EDRR is that for standard oral dosing with no transit compartments, and the four EDRR boundary curves are for transit compartment models with varying number of transit compartments *n*. **a** Varying *n* with fixed mean transit time $MTT=4.4$ h, so that $k=\frac{n}{MTT}$. **b** Varying *n* with fixed transit rate constant $k=0.45\,$h$^{-1}$, so that $MTT=\frac{n}{k}$

In Fig. 11, we show EDRRs for transit compartment models with varying number of transit compartments *n*, while fixing either the transit rate constant or the mean transit time (which effects the “smoothed delay”). In each case, the EDRR is petal-shaped. Since the smoothed delay gives a narrower band for the steady-state timecourse than pure time-lag, the transit compartment EDRRs always contain the standard oral-dosing EDRR as a subset. Increasing either the timing of the delay (by decreasing *n* with *k* fixed) or its “spread” (by increasing *n* with *MTT* fixed) results in extension of the EDRR. For safe, conservative dosing regimen design, any regimen within the standard oral-dosing EDRR can, of course, be chosen.

### Three-parameter dosing regimens

While equi-bolus dosing (input (Beq), with parameters *T* and $D_{0}$) is often of interest, we note that the cases of equi-bolus dosing with loading dose (input (BeqL)) and equi-infusion dosing (input (Ieq)) are also important clinically [43]. Appropriate three-dimensional EDRRs for these three-parameter regimens for IV administration may also be constructed using the analytical results.

#### Equi-dosing regimen region for IV equi-bolus dosing with loading dose

For model (M1) with forcing (BeqL), we seek constraints on the three equi-dosing regimen parameters $D_{0}$, *T* and $D_{L}$ such that the time course given by (3.4) gives drug level that is safe and therapeutic *immediately and always*. The peak and trough levels are monotonic in time, so the necessary and sufficient constraints are that the steady-state time course given by (3.3) has

5.7 $$\begin{aligned} D_{me}<a_{c}^{\infty }(t_{\infty })<D_{MS}, \end{aligned}$$

and also that the drug level for the first dosing interval is entirely within the therapeutic range, i.e.

5.8 $$\begin{aligned} D_{me}e^{k_{e}T}< FD_{L} < D_{MS}. \end{aligned}$$

For any given loading dose, the steady-state constraints require *T* and $D_{L}$ to be within the petal-shaped two-parameter EDRR as before. The added constraints (5.8) limit the dosing interval duration such that

$$\begin{aligned} T<\frac{1}{k_{e}}\log \frac{FD_{L}}{D_{me}}. \end{aligned}$$

**Fig. 12:**
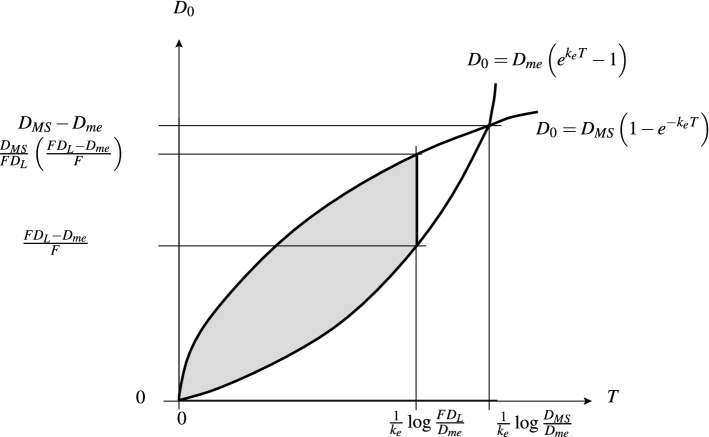
The two-dimensional equi-dosing regimen region (the shaded region) for equi-bolus $(T,D_{0})$ dosing with fixed loading dose $D_{L}$. Due to the added loading dose constraints (5.8), the slice is now a “chopped petal”

Thus, for fixed $D_{L}$, the two-parameter ($T,D_{0}$) dosing regimen region is a “chopped petal” shape, as shown in Fig. 12. The three-dimensional EDRR is given by the union of all such chopped petals, with $D_{L}=\frac{D_{me}}{F}e^{k_{e}T}$ as a boundary surface.

**Fig. 13:**
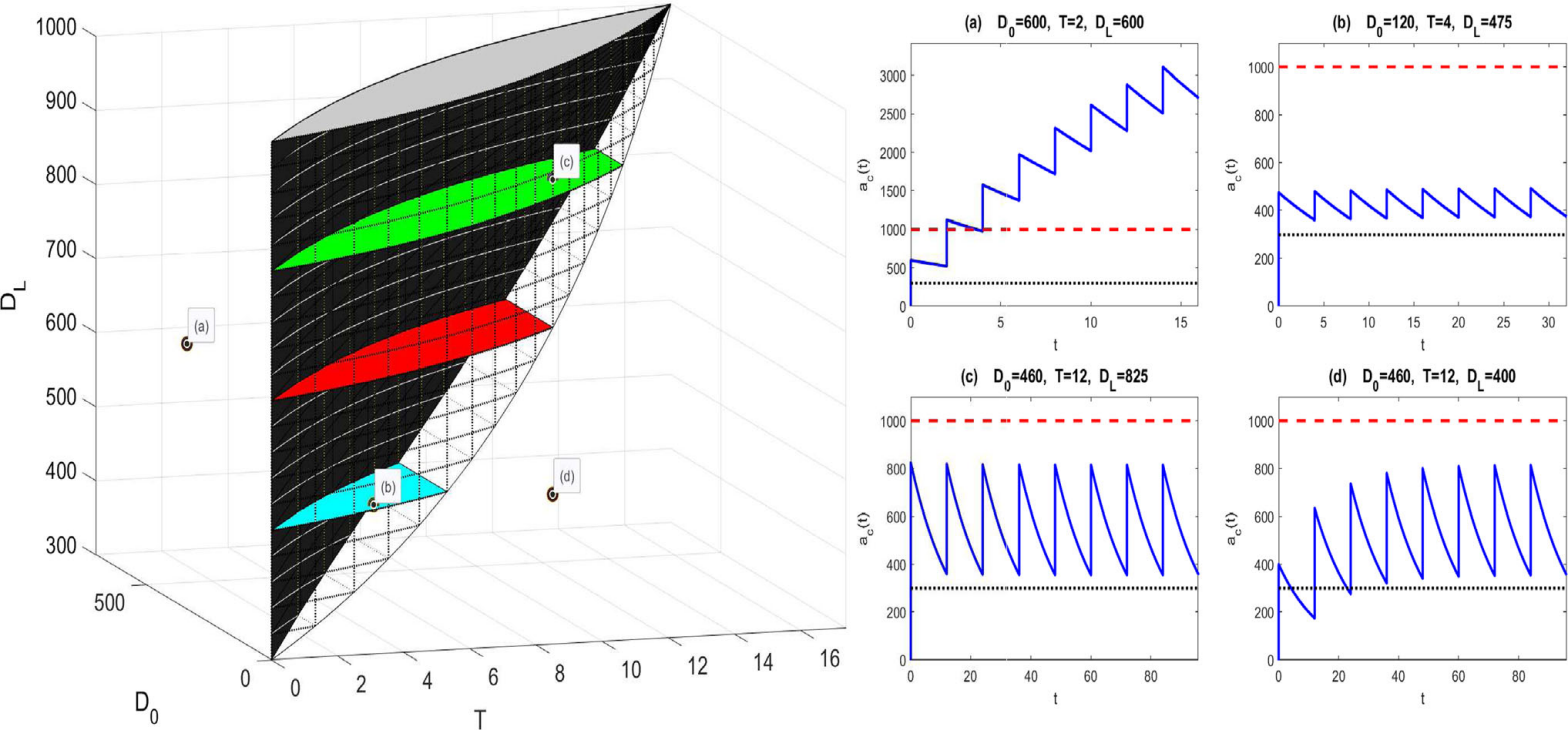
Three-dimensional equi-dosing regimen region (EDRR) for IV dosing with loading dose, with $F=1$, $k_{e}$=0.0692 h$^{-1}$, and hypothetical minimum effective and maximum safe drug levels $D_{me}=300$ mg and $D_{MS}=1000$ mg. Three “chopped petal” cross sections are highlighted for illustration: $D_{L}=475$ (blue), $D_{L}=650$ (red), $D_{L}=825$ (green). Sample time courses $a_{c}(t)$ for four $(T,D_{0},D_{L})$ regimens are shown. Only regimens (b) and (c) are within the three-parameter EDRR. Regimen (d) gives time course which is not therapeutic immediately, but it is at steady-state

A computed three-parameter EDRR with illustrative drug level time courses is shown in Fig. 13. The upper boundary surface projected onto the $(T,D_{0})$ plane gives the two-parameter EDRR for IV dosing (see further visualisation in Appendix 3). Only regimens (b) and (c) are within the three-parameter EDRR. Regimen (d) gives time course which is not therapeutic immediately, but it is at steady-state; it is clear that addition of a larger loading dose as in (c) gives an acceptable regimen. Practical dosing considerations such as frequency of administration, and therapeutic considerations such as drug level fluctuation, dosing interval averages and safety margins, can lead to specific dosing protocols [43]. Regimens following such protocols can easily be found by exploring the EDRR intuitively, for example, taking into account proximity to EDRR boundaries.

#### IV infusion equi-dosing

For model (M1) with forcing (Ieq), we have the three parameter dosing regimen $(T,k_{in},t_{f})$, and seek constraints for safe and therapeutic drug levels at steady-state. From (3.8), since the maximum and minimum drug levels at steady-state occur at $t_{\infty }=t_{f}$ and $t_{\infty }=0$ respectively, we readily find that

5.9 $$\begin{aligned} \max (a_{c}^{\infty }) = a_{c}^{\infty }(t_{f}) = \frac{Fk_{in}}{k} \left( \frac{1-e^{-k_{e}t_{f}}}{1-e^{-k_{e}T}}\right) , \end{aligned}$$

and

5.10 $$\begin{aligned} \min (a_{c}^{\infty }) = a_{c}^{\infty }(t_{f}) = \frac{Fk_{in}}{k} \left( \frac{1-e^{k_{e}t_{f}}}{1-e^{k_{e}T}}\right) . \end{aligned}$$

The acceptable three-dimensional equi-dosing regimen region (for $t_{f}<T$) is therefore given by

5.11 $$\begin{aligned} D_{me}\frac{1-e^{k_{e}T}}{1-e^{k_{e}t_{f}}}< \frac{Fk_{in}}{k_{e}} < D_{MS} \frac{1-e^{-k_{e}T}}{1-e^{-k_{e}t_{f}}}. \end{aligned}$$

**Fig. 14:**
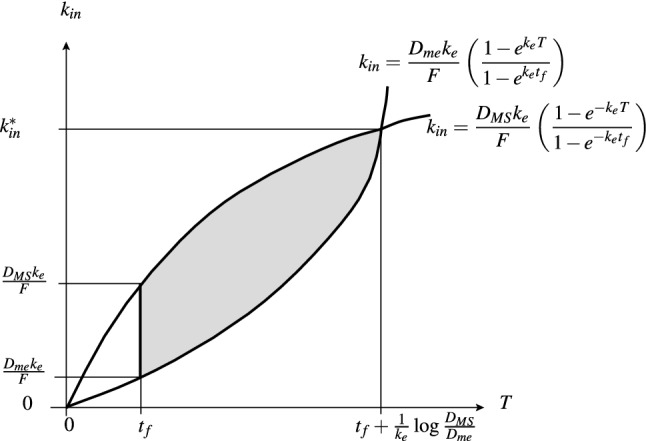
The two-dimensional equi-dosing regimen region (the shaded region) for equi-infusion with fixed $t_{f}$. The value $k_{in}^{*}$ is given in (5.12)

It is instructive first to consider a two-parameter $(T,k_{in})$ EDRR, given fixed infusion off-time $t_{f}$. We find another chopped petal two-dimensional region, now as illustrated in Fig. 14. It is straightforward to show that the cross-over value of infusion rate is

5.12 $$\begin{aligned} k_{in}^{*}=\frac{k_{e}}{F} \left( \frac{D_{MS}-D_{me}e^{-k_{e}t_{f}}}{1-e^{-k_{e}t_{f}}}\right) . \end{aligned}$$

The petal does not extend to the origin in the $(T,k_{in})$-plane; it is chopped at at $T=t_{f}$ due to the simple constraint that $t_{f}<T$.

**Fig. 15:**
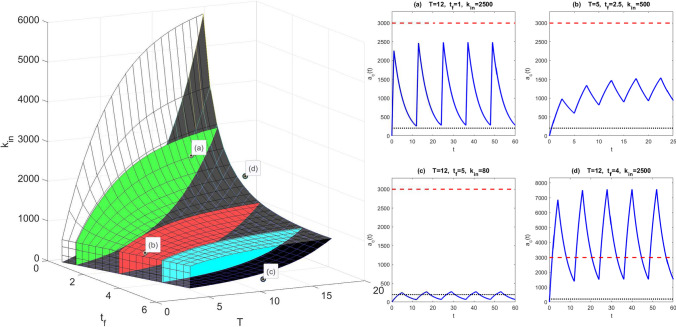
Three-dimensional $(T,t_{f},D_{0})$ equi-dosing regimen region for multi-infusion, with $F=1$, $k_{e}$=0.2$^{-1}$, and hypothetical minimum effective and maximum safe drug levels $D_{me}=200$ mg and $D_{MS}=3000$ mg. Three “chopped petal” cross sections are highlighted for illustration: $t_{f}=4$ (blue), $t_{f}=2.5$ (red), $t_{f}=1$ (green). Sample time courses $a_{c}(t)$ for four regimens are shown. Only regimens **a** and **b** are within the three-parameter EDRR (Color figure online)

The three-dimensional EDRR is given by the union of all such chopped petals as $t_{f}$ varies, bounded by the planes $T=t_{f}$ and $T=t_{f}+\frac{1}{k_{e}}\log \frac{D_{MS}}{D_{me}}$. In Fig. 15, we show a computed three-parameter EDRR for a range of $t_{f}$ values, with illustrative drug level time courses. It is clear that dosing regimens can easily be chosen from within the EDRR. Only regimens (a) and (b) are within the three-parameter EDRR. Furthermore, systematic, direction-based regimen perturbations within the EDRR can be made to affect time course properties such as fluctuation level. The EDRR is proposed as a useful summary towards dosing regimen design.

## Discussion

We have derived new analytical solutions for a generalised multi-dose transit compartment model (TCM), extending the analysis of the popular model in [44]. These solutions provide a means for analysing and parameterising delayed drug-level time courses without the need for nonsmooth time-lag models. The smoothed delay profile may be seen to give a better fit to experimental data than pure time delay models. The generalised model allows for simulation of realistic repeated dosing regimens that have traditionally been analysed in detail for simpler one- and two-compartment models [10, 43, 45]. In this sense, we importantly bridge the gap between traditional multi-dose analysis and the time-delay and TCM literature [23, 26–28, 38, 44, 55], providing a powerful method for capturing delays.

The exact solutions for the multi-dosing TCM will serve primarily as a tool for PK analysis. Further complexity may also be added as a future modelling extension by considering the “body” as a two-compartment schematic including central and peripheral compartments. Since first-order transfer is typically considered between these compartments [20, 43], the resulting ODE system including the full transit compartment cascade will be linear. As such, we expect analytical solutions to be available again, via the Laplace Transform method. Our new TCM analysis may also have application beyond PK. For example, signal transduction dynamics can often be modelled with linear transit compartment cascades [15, 30, 47, 54]. Recently, solutions comprising incomplete gamma functions have been found for linear signal transduction cascades under different input conditions [5]. We expect that further analysis of periodic impulsive inputs to such systems will be valuable.

Drug dosing regimen design is an important consideration in therapeutics, from the stage of drug development [39] through to personalised regimens [14, 36]. Given PK parameters, prediction of drug levels based on regimen parameters is common. With analytical expressions for drug levels as functions of time, we have shown that constraints on dosing parameters are readily available at the outset of any simulation-based study. Furthermore, the corresponding equi-dosing regimen regions (EDRRs) provide a novel, clear and succinct summarising visualisation of the acceptable dosing regimen parameter space, which may be explored intuitively to design effective and non-toxic treatments.

Predictive modelling using ODE models for PK is common, with end-user pharmacologists widely using exact solutions for low-dimensional compartmental models via a range of computational tools. Rapid computation of predicted time courses for multiple dosing regimens has further been facilitated by the development of used-friendly simulation packages (e.g. [16, 18, 22]). EDRR visualisation can easily be achieved through a variety of computational tools, and we suggest that EDRRs could easily be incorporated into a number of packages to aid regimen design studies.

Finally, we remark that the mathematical detail of our work is also interesting in its own right, under the banner of *mathematical pharmacology*, which is now a recognised and growing field [51]. In [29], simple compartmental PK models are proposed as a starting point for biomathematics study and research. We propose a number of model perturbations and related mathematical directions beyond the scope of the current work. Constructing exact solutions for the TCM model relies on evaluation of the lower incomplete gamma function $\gamma (n,t)$, which we have explored in some detail. An assessment of the practicality of using the analytical TCM results here versus numerical ODE solutions would be useful. Incorporation of more efficient and accurate approximation for $\gamma (n,t)$, especially for parameter estimation purposes, may be a valuable pursuit, as discussed in [1, 2, 7, 50]. We have proposed a thorough practical and sturctural identifiability analysis of our TCM (‘Transit compartments—smoothed delays, lag time and data fitting (single-dose)’ and Appendix 2.3). A theoretical comparison of the new TCM results and delay-differential equation (DDE) modelling approaches (see [26, 38]) under impulse train inputs is also warranted. Linear pharmacokinetics is studied in our work, and much of the solution method relies on time-invariance of the PK parameters. However, chronopharmacokinetics is an important phenomenon that should be considered in PK ODE modelling [8, 21, 52]. Extension of our models and methodology to incorporate time-dependent parameters will be explored in future, but may be limited to numerical computation. Further, wider applicability of the TCM and EDRR methods will be achieved by consideration of nonlinear Michaelis-Menten elimination, which is discussed mathematically in [49, 53]. Also, importantly, the PK models here may be linked to pharmacodynamics (PD) models to explore predicted drug responses; PD models are described in detail in [20, 25, 43, 45].

## Figures and Tables

**Fig. 16:**
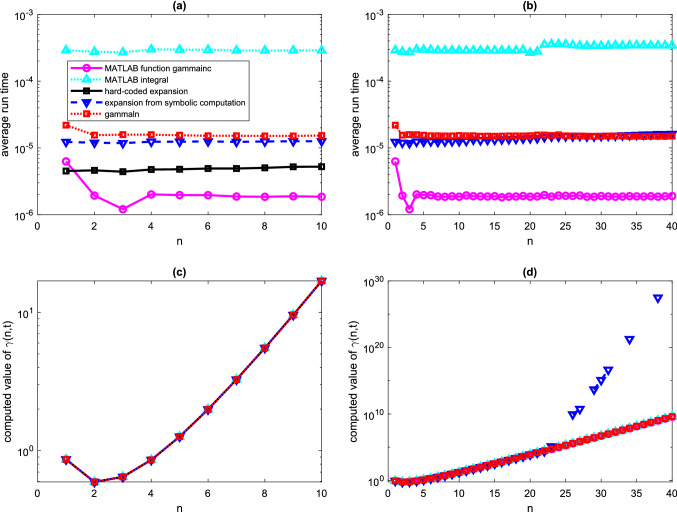
Run time (desktop PC, Intel(R) Core(TM) i5-9400, 2.9GHz) and value for different implementations of the lower incomplete gamma function $\gamma (n,t)$ in MATLAB. The methods include (i) use of built-in function gammainc, (ii) numerical evaluation of the integral in (3.17) using integral command, (iii) hard-coding the sum in the final term of (3.17) for $n=1, \ldots , 10$, (iv) use of MATLAB’s Symbolic Toolbox to expand the sum, (v) use of built-in functions gammaln and gamcdf for log-gamma and cumulative distribution functions for the calculation in (3.32). A typical result is shown, taking $t=2$. Similar results are seen for a range of *t* values. The average evaluation time in seconds is found using MATLAB’s timeit command. **a** Evaluation times for different methods, for $n=1, \ldots , 10$. **b** Evaluation times for different methods, for $n=1, \ldots , 40$. **c** Computed values for different methods, for $n=1, \ldots , 10$. **d** Computed values for different methods, for $n=1, \ldots , 40$

**Fig. 17:**
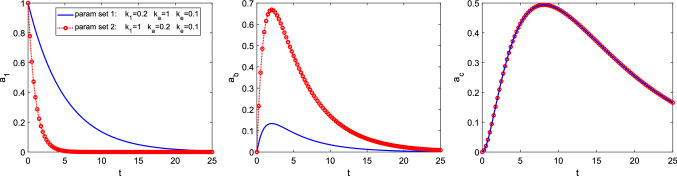
Non-identifiability of *k* and $k_{a}$ for transit compartment model with $n=1$. Solutions for a single bolus dose with $FD_{0}=1$ are shown, for two parameter sets. Parameter set 1 has $(k,k_{a},k_{e})=(0.2,1,0.1)$, and parameter set 2 has $(k,k_{a},k_{e})=(1,0.2,0.1)$. The two parameter sets lead to identical time course for the observed output $a_{c}(t)$, via different time courses for $a_{1}(t)$ and $a_{b}(t)$

**Fig. 18:**
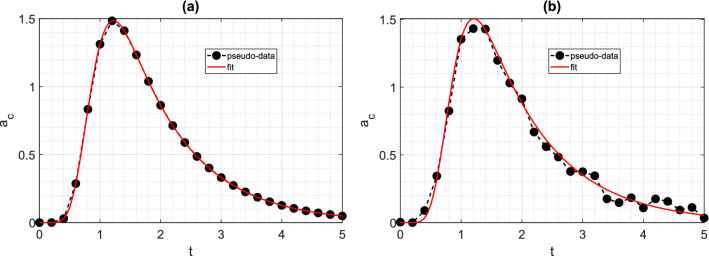
Parameter fitting for transit compartment model with $n=10$. Pseudo-data (time courses of $a_{c}(t)$) are generated by simulating with base parameter values $k=12.76\,$h$^{-1}$, $k_{a}=9.11\,$h$^{-1}$, $k_{e}=0.96\,$h$^{-1}$, $F=0.69$, and a single bolus dose of 3.5 mg. **a** No noise is added to the simulated time course, so the data are “perfect”. The four parameter values are estimated perfectly. **b** Randomly generated noise (with an upper bound of 10% of the simulated peak value) is added to create noisy data. The parameter estimates returned are $k=13.45\,$h$^{-1}$, $k_{a}=7.46\,$h$^{-1}$, $k_{e}=0.92\,$h$^{-1}$, $F=0.67$

**Fig. 19:**
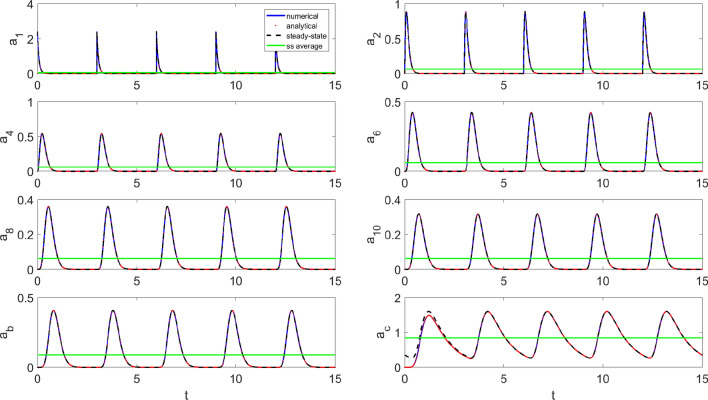
Equi-dosing time courses for transit compartment model (Mt,Beq), showing transit, absorption and central compartment drug levels. Simulated time courses (Mt,Beq) for $n=10$ transit compartments with $MTT=0.7837$h. Here, $F=0.69$, $k_{e}$=0.96 h$^{-1}$ $k_{a}$=9.11 h$^{-1}$, *k*=12.76 h$^{-1}$. Equi-dosing regimen has $D_{0}$=3.5 mg, *T*=3h. For validation, we show results from analytical solution and a numerically computed solution. The dosing interval average at steady-state is indicated. Also, the steady-state profile is shown for each compartment

**Fig. 20:**
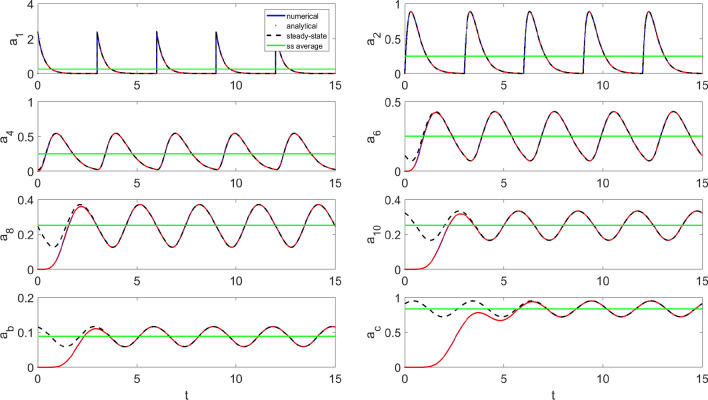
Equi-dosing time courses for transit compartment model (Mt,Beq), showing transit, absorption and central compartment drug levels. Simulated time courses (Mt,Beq) for $n=10$ transit compartments with $MTT=0.7837$h. Here, $F=0.69$, $k_{e}$=0.96 h$^{-1}$ $k_{a}$=9.11 h$^{-1}$, *k*=3.19$\,h^{-1}$. Equi-dosing regimen has $D_{0}$=3.5 mg, *T*=3 h. For validation, we show results from analytical solution and a numerically computed solution. The dosing interval average at steady-state is indicated. Also, the steady-state profile is shown for each compartment

**Fig. 21:**
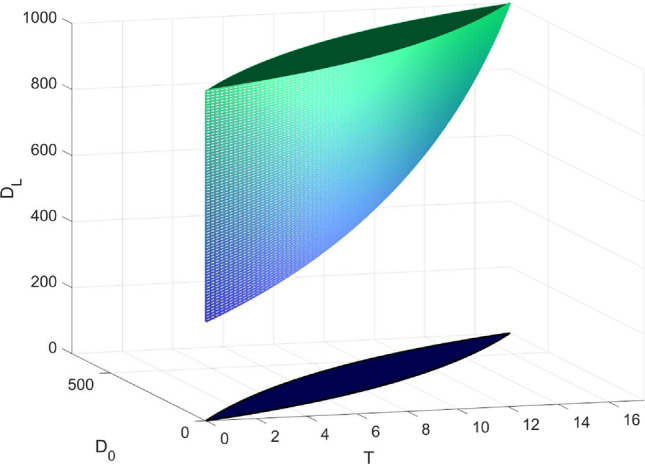
Three-dimensional $(T,D_{0},D_{L})$ equi-dosing regimen region (EDRR) for IV dosing with loading dose, with $F=1$, $k_{e}$=0.0692 h$^{-1}$, and hypothetical minimum effective and maximum safe drug levels $D_{me}=300$ mg and $D_{MS}=1000$ mg, as in Fig. 13. Projection onto $(T,D_{0})$-plane (the shadow) is the standard two-parameter full-petal EDRR for **steady-state** conditions for oral dosing. The shadow of any chopped petal slice ($D_{L}=$ constant) through the three-dimensional EDRR is contained within this

## Appendix 1: multi-dosing solutions

Equi-dosing solutions are derived here. While the results are well known for the one-compartment and two-compartment models, it is instructive to see their solutions derived by Laplace Transform methods prior to deriving the full transit compartment model solution. Further, the steady-state solutions are vital for construction of EDRRs.

### Single compartment—equi-bolus dosing

The solution to the (M1,Beq) problem (see Table 1) can easily be found using Laplace Transforms. We take the Laplace Transform of the IVP, giving

and hence

A.1 $$\begin{aligned} A_{c}(s)=\frac{G_{B}(s)}{s+k_{e}}. \end{aligned}$$

For dosing regimen (Beq), the Laplace Transform of the dosing rate (2.3) is

A.2 $$\begin{aligned} G_{B}(s)=FD_{0}\sum _{j=1}^{M}e^{-(j-1)Ts}, \end{aligned}$$

and hence

$$\begin{aligned} A_{c}(s)=FD_{0}\sum _{j=1}^{M} \frac{e^{-(j-1)Ts}}{s+k_{e}}. \end{aligned}$$

Taking the inverse Laplace Transform, we find the solution for the drug amount in the central compartment as

A.3 $$\begin{aligned} a_{c}(t)=FD_{0}\sum _{j=1}^{M} H\big (t-(j-1)T\big ) e^{-k_{e}\big (t-(j-1)T\big )}, \end{aligned}$$

where *H* is the Heaviside function. Since

A.4 $$\begin{aligned} t_{j}=t-(j-1)T = \text { time since j}^{th} \text { dose}, \end{aligned}$$

we can write

A.5 $$\begin{aligned} a_{c}(t)=FD_{0}\sum _{j=1}^{M} H\big (t_{j}) e^{-k_{e}(t_{j})}. \end{aligned}$$

This solution may be written compactly without summation notation by considering, for example, the central compartment drug level after the *M*th dose, $a_{c}^{M}(t_{M})$. The finite geometric series resulting from (3.1) may be evaluated to give

A.6 $$\begin{aligned} a_{c}^{M}(t_{M}) = FD_{0}\left( \frac{1-e^{-Mk_{e}T}}{1-e^{-k_{e}T}}\right) e^{-k_{e} t_{M}}, \;\; \text {for } 0\le t_{M} <T. \end{aligned}$$

We readily find the steady-state (*T*-periodic) behaviour by letting $M\rightarrow \infty$ in (A.6), giving

A.7 $$\begin{aligned} a_{c}^{\infty }(t_{\infty })=\left( \frac{FD_{0}}{1-e^{-k_{e}T}}\right) e^{-k_{e} t_{\infty } }, \;\; \text {for } 0\le t_{\infty } <T. \end{aligned}$$

For model (M1) with forcing (BeqL), superposition of (3.1) with the solution corresponding to a single extra bolus input of $(D_{L}-D_{0})$ at time $t=0$ gives the solution for the central compartment drug level as

A.8 $$\begin{aligned} a_{c}(t) = F \left\{ D_{0}\left( \sum _{j=1}^{M} H\big (t_{j}) e^{-k_{e}t_{j}}\right) + (D_{L}-D_{0})e^{-k_{e}t}\right\} . \end{aligned}$$

The central compartment drug level after the *M*th dose, $a_{c}^{M}(t_{M})$, is then given by

A.9 $$\begin{aligned}&a_{c}^{M}(t_{M}) = F \left\{ D_{0}\left( \frac{1-e^{-Mk_{e}T}}{1-e^{-k_{e}T}}\right) e^{-k_{e} t_{M}}\right. \nonumber \\&\left. \quad + (D_{L}-D_{0})e^{-k_{e}(t_{M}+(M-1)T)} \right\} . \end{aligned}$$

The loading dose effect is transient, so the steady-state behaviour is unaffected by $D_{L}$.

### Single compartment—equi-infusion dosing

The solution to the (M1,Ieq) problem (see Table 1) can be found using Laplace Transforms. Again, taking the Laplace Transform of the IVP gives

and hence

A.10 $$\begin{aligned} A_{c}(s)=\frac{G_{I}(s)}{s+k_{e}}. \end{aligned}$$

For dosing regimen (Ieq), the Laplace Transform of the dosing rate (2.5) is

A.11 $$\begin{aligned} G_{I}(s)=Fk_{in}\sum _{j=1}^{M} \frac{e^{-(j-1)Ts}-e^{-((j-1)T+t_{f})s}}{s}, \end{aligned}$$

and hence

$$\begin{aligned} A_{c}(s)=Fk_{in}\sum _{j=1}^{M} \frac{e^{-(j-1)Ts}-e^{-((j-1)T+t_{f})s}}{s(s+k_{e})}. \end{aligned}$$

Using the inverse Laplace Transform result [56]

$$\begin{aligned} {\mathcal {L}}^{-1}\left\{ \frac{e^{-as}}{s(s+k_{e})}\right\} = H(t-a)(1-e^{-k_{e}(t-a)}), \end{aligned}$$

we find the solution for the drug amount in the central compartment as

A.12 $$\begin{aligned} a_{c}(t)= \frac{Fk_{in}}{k_{e}} \sum _{j=1}^{M} H(t_{j})(1-e^{-k_{e}t_{j}})- H(t_{j}-t_{f})(1-e^{-k_{e}(t_{j}-t_{f})}), \end{aligned}$$

where $t_{j}=t-(j-1)T$. Again, a geometric series results, and for $0\le t_{M} <T$, we may express the drug level as

A.13 $$\begin{aligned}&a_{c}^{M}(t_{M}) = \frac{Fk_{in}}{k_{e}} \left\{ \underbrace{(1-e^{-k_{e}t_{M}}) - H(t_{M}-t_{f})(1-e^{-k_{e}(t_{M}-t_{f})})}_{\text {contribution from}} M\text {th} \text {dose}\right. \nonumber \\&\left. \quad + \underbrace{ (e^{k_{e}t_{f}}-1) \left( \frac{e^{-Mk_{e}T}-e^{-k_{e}T}}{e^{-k_{e}T}-1}\right) e^{-k_{e}t_{M}} }_{\text {accumulation}} \right\} \; . \end{aligned}$$

Letting $M\rightarrow \infty$, we see that the steady-state (*T*-periodic) level is given by

A.14 $$\begin{aligned} a_{c}^{\infty }(t_{\infty })= & {} \frac{Fk_{in}}{k_{e}} \left\{ 1-\frac{e^{k_{e}t_{f}}-e^{k_{e}T}}{1-e^{k_{e}T}} e^{-k_{e}t_{\infty }}\right. \nonumber \\&\left. -H(t_{\infty }-t_{f})(1-e^{-k_{e}(t_{\infty }-t_{f})}) \right\} , \;\; \text {for } 0\le t_{\infty } <T. \end{aligned}$$

Alternatively, we may express this as

A.15 $$\begin{aligned} a_{c}^{\infty }(t_{\infty }) = \frac{Fk_{in}}{k_{e}} \left\{ \frac{e^{k_{e}T}-e^{k_{e}t_{f}}}{1-e^{k_{e}T}} e^{-k_{e}t_{\infty }} + e^{-k_{e}(t_{\infty }-t_{f})H(t_{\infty }-t_{f})} \right\} . \end{aligned}$$

We note here that the standard result (see, e.g., [45]) for a single continuous infusion is found by taking $M=1$ in (3.6) to give

A.16 $$\begin{aligned} a_{c}(t)=\frac{Fk_{in}}{k_{e}}(1-e^{-k_{e}t}). \end{aligned}$$

### Two compartments—equi-bolus dosing

With a view to generalising the model to multiple transit compartment absorption, it is convenient to write the system (M2) (from Table 1) in matrix form as

A.17a $$\begin{aligned} \frac{d}{dt}\underline{\mathbf {x}} = B\underline{\mathbf {x}} + \underline{\mathbf {g}}, \qquad \underline{\mathbf {x}}(0)=\underline{\mathbf {0}}, \end{aligned}$$

where

A.17b $$\begin{aligned} \underline{\mathbf {x}} = \begin{pmatrix} a_{b}(t) \\ a_{c}(t) \end{pmatrix}, \;\;\; B=\begin{pmatrix} -k_{a} &{} 0 \\ k_{a} &{} -k_{e} \end{pmatrix}, \;\;\; \underline{\mathbf {g}}=\begin{pmatrix}g_{B}(t) \\ 0 \end{pmatrix} \; . \end{aligned}$$

We solve the matrix ODE problem again using the method of Laplace Transforms. Firstly, we have

$$\begin{aligned} s\underline{\mathbf {X}}(s) = B\underline{\mathbf {X}}(s) + \begin{pmatrix} G_{B}(s) \\ 0 \end{pmatrix}, \end{aligned}$$

which gives

A.18 $$\begin{aligned} \underline{\mathbf {X}}(s) = (sI-B)^{-1}\begin{pmatrix} G_{B}(s) \\ 0 \end{pmatrix}, \end{aligned}$$

We readily find

$$\begin{aligned} (sI-B)^{-1} = \frac{1}{(s+k_{a})(s+k_{e})}\begin{pmatrix} s+k_{e} &{} 0 \\ k_{a} &{} s+k_{a} \end{pmatrix}, \end{aligned}$$

so that the Laplace Transform of the solution is

A.19 $$\begin{aligned} \underline{\mathbf {X}}(s) = \begin{pmatrix} \frac{G_{B}(s)}{s+k_{a}} \\ \frac{G_{B}(s)}{(s+k_{a})(s+k_{e})} \end{pmatrix} = \begin{pmatrix} \frac{G_{B}(s)}{s+k_{a}} \\ \frac{G_{B}(s)}{k_{e}-k_{a}} \left( \frac{1}{s+k_{a}} - \frac{1}{s+k_{e}} \right) \end{pmatrix} . \end{aligned}$$

Taking the inverse Laplace Transform, we find the solution

A.20 $$\begin{aligned} \underline{\mathbf {x}}(t) = \begin{pmatrix} a_{b}(t) \\ a_{c}(t) \end{pmatrix} = \begin{pmatrix} FD_{0}\sum _{j=1}^{M} H(t_{j}) e^{-k_{a}t_{j}} \\ \frac{k_{a}}{k_{e}-k_{a}} FD_{0}\sum _{j=1}^{M} H(t_{j}) \left[ e^{-k_{a}t_{j}} - e^{-k_{e}t_{j}}\right] \end{pmatrix} \; . \end{aligned}$$

### Transit compartments—equi-bolus dosing

The solution to the (Mt,Beq) problem (see Table 1) can be found using Laplace Transforms. Taking the Laplace Transform of the IVP (2.6) (with $g_{1}(t)=g_{B}(t)$) gives

A.21 $$\begin{aligned} (sI-B)\underline{\mathbf {X}}(s) = \underline{\mathbf {G}}(s), \end{aligned}$$

where the only nonzero element of $\underline{\mathbf {G}}(s)$ is $G_{1}(s)=G_{B}(s)$. Now, $(sI-B)$ is lower bidiagnoal, given by:

A.22 $$\begin{aligned} (sI-B) = \left( \begin{array}{ccccccc} s+k &{} &{} &{} &{} &{} &{} \\ -k &{} s+k &{} &{} &{} &{} &{} \\ &{} -k &{} s+k &{} &{} &{} &{} \\ &{} &{} \ddots &{} \ddots &{} &{} &{} \\ &{} &{} &{} -k &{} s+k &{} &{} \\ &{} &{} &{} &{} -k &{} s+k_{a} &{} \\ &{} &{} &{} &{} &{} -k_{a} &{} s+k_{e} \end{array} \right) \; . \end{aligned}$$

Hence (A.21) may be solved easily by a process of forward substitution, or by noting that $(sI-B)^{-1}$ is lower triangular, given by:

A.23 $$\begin{aligned} (sI-B)^{-1} = \left( \begin{array}{ccccccc} \frac{1}{s+k} &{} &{} &{} &{} &{} &{} \\ \frac{k}{(s+k)^{2}} &{} \frac{1}{s+k} &{} &{} &{} &{} &{} \\ \frac{k^{2}}{(s+k)^{3}} &{} \frac{k}{(s+k)^{2}} &{} \frac{1}{s+k} &{} &{} &{} &{} \\ \vdots &{} \vdots &{} \vdots &{} \ddots &{} &{} &{} \\ \frac{k^{n-1}}{(s+k)^{n}} &{} \frac{k^{n-2}}{(s+k)^{n-1}} &{} \frac{k^{n-3}}{(s+k)^{n-2}} &{} \cdots &{} \frac{1}{s+k} &{} &{} \\ \frac{k^{n}}{(s+k)^{n}(s+k_{a})} &{} \frac{k^{n-1}}{(s+k)^{n-1}(s+k_{a})} &{} \frac{k^{n-2}}{(s+k)^{n-2}(s+k_{a})} &{} \cdots &{} \frac{k}{(s+k)(s+k_{a})} &{} \frac{1}{s+k_{a}} &{} \\ \frac{k^{n}k_{a}}{(s+k)^{n}(s+k_{a})(s+k_{e})} &{} \frac{k^{n-1}k_{a}}{(s+k)^{n-1}(s+k_{a})(s+k_{e})} &{} \frac{k^{n-2}k_{a}}{(s+k)^{n-2}(s+k_{a})(s+k_{e})} &{} \cdots &{} \frac{k k_{a}}{(s+k)(s+k_{a})(s+k_{e})} &{} \frac{k_{a}}{(s+k_{a})(s+k_{e})} &{} \frac{1}{s+k_{e}} \\ \end{array} \right) \; . \end{aligned}$$

The Laplace Transform of the drug level $a_{i}(t)$ in the $i^\text {th}$ transit compartment is then given by

A.24 $$\begin{aligned}&A_{i}(s) \;\; = \;\; (sI-B)^{-1}_{1,i} \; G_{1}(s)\nonumber \\&\quad \;\; = \;\; \frac{k^{i-1}}{(s+k)^{i}} \times G_{B}(s) \qquad \text {for } i=1, \ldots , n \; . \end{aligned}$$

The Laplace Transform of the drug level $a_{b}(t)$ in the absorption compartment is

A.25 $$\begin{aligned}&A_{b}(s) \;\; = \;\; (sI-B)^{-1}_{1,n+1} \; G_{1}(s)\nonumber \\&\quad \;\; = \;\; \frac{k^{n}}{(s+k)^{n}(s+k_{a})} \times G_{B}(s) , \end{aligned}$$

while the transform of the drug level $a_{c}(t)$ in the central compartment is

A.26 $$\begin{aligned}&A_{c}(s) \;\; = \;\; (sI-B)^{-1}_{1,n+2} \; G_{B}(s)\nonumber \\&\quad \;\; = \;\; \frac{ k^{n} k_{a}}{(s+k)^{n}(s+k_{a})(s+k_{e})} \times G_{B}(s) , \end{aligned}$$

where, as before,

A.27 $$\begin{aligned} G_{B}(s)=FD_{0}\sum _{j=1}^{M}e^{-(j-1)Ts}. \end{aligned}$$

For the transit compartments, we find from (A.24) and (A.27) that

$$\begin{aligned} a_{i}(t) \;\; = \;\; {\mathcal {L}}^{-1} \left\{ A_{i}(s)\right\} \;\; = \;\; FD_{0} k^{i-1} {\mathcal {L}}^{-1} \left\{ \sum _{j=1}^{M} \frac{e^{-(j-1)Ts}}{(s+k)^{i}} \right\} . \end{aligned}$$

Now, given that

A.28 $$\begin{aligned}&{\mathcal {L}}^{-1} \left\{ \frac{e^{-(j-1)Ts}}{(s+k)^{i}}\right\} \nonumber \\&\quad = \frac{1}{(i-1)!} \big (t-(j-1)T\big )^{i-1} e^{-k\big (t-(j-1)T\big )} H\big (t-(j-1)T\big ), \nonumber \\&\quad = \frac{1}{(i-1)!} t_{j}^{i-1} e^{-kt_{j}} H(t_{j}), \qquad \text {where} \;\; t_{j}=t-(j-1)T, \end{aligned}$$

we find that

A.29 $$\begin{aligned} \boxed {a_{i}(t) \;\; = \;\; \frac{F D_{0}k^{i-1}}{(i-1)!} \sum _{j=1}^{M} H(t_{j}) t_{j}^{i-1} e^{-kt_{j}}, \qquad i=1, \ldots , n.} \end{aligned}$$

For the absorption compartment, we find from (A.25) and (A.27) that

A.30 $$\begin{aligned} a_{b}(t) = FD_{0}k^{n} {\mathcal {L}}^{-1} \left\{ \sum _{j=1}^{M} \frac{e^{-(j-1)Ts}}{(s+k)^{n}} \times \frac{1}{s+k_{a}} \right\} . \end{aligned}$$

Now,

A.31a $$\begin{aligned} {\mathcal {L}}^{-1} \left\{ \frac{1}{s+k_{a}}\right\} = e^{-k_{a}t}H(t), \end{aligned}$$

and, from (A.28),

A.31b $$\begin{aligned} {\mathcal {L}}^{-1} \left\{ \frac{e^{-(j-1)Ts}}{(s+k)^{n}}\right\} = \frac{1}{(n-1)!} t_{j}^{n-1} e^{-kt_{j}} H(t_{j}). \end{aligned}$$

Setting $\tau _{j}=\tau -(j-1)T$, we readily compute the following inverse transform as a convolution:

A.31c $$\begin{aligned}&{\mathcal {L}}^{-1} \left\{ \frac{e^{-(j-1)Ts}}{(s+k)^{n}} \times \frac{1}{s+k_{a}} \right\} = \int _{0}^{t} \left[ \frac{1}{(n-1)!} \tau _{j}^{n-1}e^{-k\tau _{j}}H(\tau _{j}) \right] \nonumber \\&\qquad \times \left[ e^{-k_{a}(t-\tau )}H(t-\tau ) \right] \; d\tau , \nonumber \\&\quad = \frac{e^{-k_{a}t}}{(n-1)!} \int _{0}^{t} H(\tau _{j}) \tau _{j}^{n-1}e^{-k\tau _{j}} e^{k_{a}\tau } \; d\tau , \nonumber \\&\quad = \frac{e^{-k_{a}t}}{(n-1)!} \int _{-(j-1)T}^{t-(j-1)T} H(\tau _{j}) \tau _{j}^{n-1}e^{-k\tau _{j}} e^{k_{a}(\tau _{j}+(j-1)T)} \; d\tau _{j}, \nonumber \\&\quad = \frac{e^{-k_{a}(t-(j-1)T)}}{(n-1)!} \int _{-(j-1)T}^{t-(j-1)T} H(\tau _{j}) \tau _{j}^{n-1}e^{-(k-k_{a})\tau _{j}} \; d\tau _{j}, \nonumber \\&\quad = \frac{H(t_{j})e^{-k_{a}t_{j}}}{(n-1)!} \int _{0}^{t_{j}} \tau _{j}^{n-1}e^{-(k-k_{a})\tau _{j}} \; d\tau _{j}. \end{aligned}$$

For $k\ne k_{a}$, we recognise the integral in the line above as one related to the lower incomplete gamma function. In particular, the lower incomplete gamma function $\gamma$, defined by (for positive integer *n*, see [3])

A.32 $$\begin{aligned} \gamma (n,t) \;\; = \;\; \int _{0}^{t} x^{n-1}e^{-x} \; dx \; \;\; = \;\; (n-1)! \left( 1-e^{-t}\sum _{p=0}^{n-1} \frac{t^{p}}{p!} \right) , \end{aligned}$$

has the following property (seen by making a change of variables $X=ax$):

A.33 $$\begin{aligned} \int _{0}^{t} x^{n-1} e^{-ax} \; dx \;\; = \;\; \frac{1}{a^{n}} \, \gamma (n,at). \end{aligned}$$

So (A.31c) gives

A.34 $$\begin{aligned} {\mathcal {L}}^{-1} \left\{ \frac{e^{-(j-1)Ts}}{(s+k)^{n}} \times \frac{1}{s+k_{a}} \right\} = \frac{H(t_{j})e^{-k_{a}t_{j}}}{(n-1)!(k-k_{a})^{n}} \gamma (n,(k-k_{a})t_{j}), \end{aligned}$$

and then (A.30) gives

A.35 $$\begin{aligned} \boxed {a_{b}(t) = \frac{F D_0}{(n-1)!} \left( \frac{k}{k-k_{a}}\right) ^{n} \sum _{j=1}^{M} H(t_{j}) e^{-k_{a}t_{j}} \, \gamma \big (n,(k-k_{a})t_{j}\big ).} \end{aligned}$$

For the central compartment, we find from (A.26) and (A.27) that

A.36 $$\begin{aligned} a_{c}(t) = FD_{0}k^{n}k_{a} {\mathcal {L}}^{-1} \left\{ \sum _{j=1}^{M} \frac{e^{-(j-1)Ts}}{(s+k)^{n}} \times \frac{1}{(s+k_{a})(s+k_{e})} \right\} . \end{aligned}$$

Now, for $k_{e}\ne k_{a}$, we have

A.37a $$\begin{aligned} {\mathcal {L}}^{-1} \left\{ \frac{1}{(s+k_{a})(s+k_{e})}\right\} = \frac{H(t)}{k_{e}-k_{a}} \left( e^{-k_{a}t}-e^{-k_{e}t}\right) , \end{aligned}$$

A.37b $$\begin{aligned}&{\mathcal {L}}^{-1} \left\{ \frac{e^{-(j-1)Ts}}{(s+k)^{n}} \times \frac{1}{(s+k_{a})(s+k_{e})} \right\} \nonumber \\&\quad = \int _{0}^{t} \left[ \frac{1}{(n-1)!} \tau _{j}^{n-1}e^{-k\tau _{j}}H(\tau _{j}) \right] \nonumber \\&\qquad \times \left[ \frac{H(t-\tau )}{k_{e}-k_{a}} \left( e^{-k_{a}(t-\tau )}-e^{-k_{e}(t-\tau )}\right) \right] \; d\tau , \nonumber \\&\quad = \frac{1}{(n-1)!(k_{e}-k_{a})}\nonumber \\&\qquad \int _{0}^{t} H(\tau _{j}) \tau _{j}^{n-1}e^{-k\tau _{j}} \left( e^{-k_{a}(t-\tau )}-e^{-k_{e}(t-\tau )}\right) \; d\tau , \nonumber \\&\quad = \frac{1}{(n-1)!} \int _{-(j-1)T}^{t-(j-1)T} H(\tau _{j}) \tau _{j}^{n-1}e^{-k\tau _{j}} \left( e^{-k_{a}t}e^{k_{a}\tau _{j}+(j-1)T}\right. \nonumber \\&\left. \qquad - e^{-k_{e}t}e^{k_{e}\tau _{j}+(j-1)T}\right) \; d\tau _{j}, \nonumber \\&\quad = \frac{H(t_{j})}{(n-1)!} \int _{0}^{t_{j}} \tau _{j}^{n-1}e^{-k\tau _{j}}\nonumber \\&\qquad \left( e^{-k_{a}t}e^{k_{a}\tau _{j}+(j-1)T} - e^{-k_{e}t}e^{k_{e}\tau _{j}+(j-1)T}\right) \; d\tau _{j}, \nonumber \\&\quad = \frac{H(t_{j})}{(n-1)!} \left\{ e^{-k_{a}(t-(j-1)T)} \int _{0}^{t_{j}} \tau _{j}^{n-1}e^{-(k-k_{a})\tau _{j}} \; d\tau _{j} \;\right. \nonumber \\&\left. \qquad - \; e^{-k_{e}(t-(j-1)T)} \int _{0}^{t_{j}} \tau _{j}^{n-1}e^{-(k-k_{e})\tau _{j}} \; d\tau _{j} \right\} , \nonumber \\&\quad = \frac{H(t_{j})}{(n-1)!} \left\{ e^{-k_{a}t_{j}} \int _{0}^{t_{j}} \tau _{j}^{n-1}e^{-(k-k_{a})\tau _{j}} \; d\tau _{j} \;\right. \nonumber \\&\left. \qquad - \; e^{-k_{e}t_{j}} \int _{0}^{t_{j}} \tau _{j}^{n-1}e^{-(k-k_{e})\tau _{j}} \; d\tau _{j} \right\} . \end{aligned}$$

Again, we write this result in terms of the lower incomplete gamma function, giving

A.38 $$\begin{aligned}&{\mathcal {L}}^{-1} \left\{ \frac{e^{-(j-1)Ts}}{(s+k)^{n}} \times \frac{1}{(s+k_{a})(s+k_{e})} \right\} \nonumber \\&\quad = \frac{H(t_{j})}{(n-1)!} \left\{ e^{-k_{a}t_{j}} \gamma (n,(k-k_{a})t) \; - \; e^{-k_{e}t_{j}} \gamma (n,(k-k_{e})t) \right\} . \end{aligned}$$

Then (A.36) gives

A.39 $$\begin{aligned} \boxed {a_{c}(t) \;\; = \;\; \frac{FD_{0}k^{n}k_{a}}{(n-1)!(k_{e}-k_{a})} \sum _{j=1}^{M} H\left( t_{j}\right) \, \left\{ \frac{e^{-k_{a}t_{j}}}{(k-k_{a})^{n}} \gamma \big (n,(k-k_{a})t_{j}\big ) \; - \; \frac{e^{-k_{e}t_{j}}}{(k-k_{e})^{n}} \gamma \big (n,(k-k_{e})t_{j}\big ) \right\} . } \end{aligned}$$

#### Steady-state behaviour

To complete the analysis (and with a view to constructing EDRR’s), we require solutions for the steady-state time course profiles. For each of the transit compartments ($i=1, \ldots , n$), one approach is to solve the ODE for $0\le t_{\infty } <T$, to give a solution parameterised by the initial value $a_{i}^{\infty }(0)$ (where $t_{\infty }=0$ corresponds to the timing of the bolus input to $a_{1}$), then use periodicity to determine the appropriate value of $a_{i}^{\infty }(0)$. In the following, we use lower case for the state variables and upper case for Laplace Transforms. For clarity and illustration, we derive the solutions for the first five transit compartments before writing the solution for transit compartment *n* in general.

For ${{compartment}\, {i=1}}$, we have

A.40a $$\begin{aligned} \frac{da_{1}^{\infty }(t_{\infty })}{dt_{\infty }}&=-k a_{1}^{\infty }(t_{\infty }), \nonumber \\ \;\; \Rightarrow \;\; s A_{1}^{\infty }(s) -a_{1}^{\infty }(0)&= -k A_{1}^{\infty }(s), \nonumber \\ \;\; \Rightarrow \;\; A_{1}^{\infty }(s)&= \frac{a_{1}^{\infty }(0)}{s+k}, \end{aligned}$$

A.40b $$\begin{aligned} \;\; \Rightarrow \;\; a_{1}^{\infty }(t_{\infty })&= a_{1}^{\infty }(0) e^{-k t_{\infty }}. \end{aligned}$$

To determine $a_{1}^{\infty }(0)$, we use the fact that there is periodic bolus dose to this compartment:

A.40c $$\begin{aligned} a_{1}^{\infty }(0)&= a_{1}^{\infty }(T)+ FD_{0}, \nonumber \\ \;\; \Rightarrow \;\; a_{1}^{\infty }(0)&= a_{1}^{\infty }(0) e^{-k T} + FD_{0}, \nonumber \\ \;\; \Rightarrow \;\; a_{1}^{\infty }(0)&= \frac{FD_{0}}{1-e^{-k T}}. \end{aligned}$$

Letting

A.41 $$\begin{aligned} \boxed {\phi =k T}, \end{aligned}$$

we write

A.42 $$\begin{aligned} a_{1}^{\infty }(0) = \frac{FD_{0}}{1-e^{-\phi }}. \end{aligned}$$

For ${{compartment}\, i=2}$, we have

A.43a $$\begin{aligned} \frac{da_{2}^{\infty }(t_{\infty })}{dt_{\infty }}&= k a_{1}^{\infty }(t_{\infty }) - k a_{2}^{\infty }(t_{\infty }), \nonumber \\ \;\; \Rightarrow \;\; s A_{2}^{\infty }(s) -a_{2}^{\infty }(0)&= k A_{1}^{\infty }(s) -k A_{2}^{\infty }(s), \nonumber \\ \;\; \Rightarrow \;\; A_{2}^{\infty }(s)&= \frac{a_{2}^{\infty }(0) + k A_{1}^{\infty }(s)}{s+k}, \nonumber \\&= \frac{a_{2}^{\infty }(0)}{s+k} + \frac{k a_{1}^{\infty }(0)}{(s+k)^{2}}, \end{aligned}$$

A.43b $$\begin{aligned} \;\; \Rightarrow \;\; a_{2}^{\infty }(t_{\infty })&= \left( a_{2}^{\infty }(0) + \frac{k a_{1}^{\infty }(0)}{1!}\, t_{\infty }\right) e^{-k t_{\infty }}. \end{aligned}$$

To determine $a_{2}^{\infty }(0)$, we use periodicity (noting that for $i>1$ the drug level is continuous):

A.43c $$\begin{aligned} a_{2}^{\infty }(0)&= a_{2}^{\infty }(T), \nonumber \\ \;\; \Rightarrow \;\; a_{2}^{\infty }(0)&= a_{2}^{\infty }(0) e^{-k T} + \frac{k a_{1}^{\infty }(0)}{1!} \, T e^{-k T}, \nonumber \\&= a_{2}^{\infty }(0) e^{-\phi } + \frac{a_{1}^{\infty }(0)}{1!} \, \phi e^{-\phi }, \end{aligned}$$

A.43d $$\begin{aligned} \;\; \Rightarrow \;\; a_{2}^{\infty }(0)&= \frac{e^{-\phi }}{1-e^{-\phi }} \times \frac{a_{1}^{\infty }(0)}{1!}\, \phi . \end{aligned}$$

Letting

A.44 $$\begin{aligned} \beta =\frac{e^{-\phi }}{1-e^{-\phi }}, \end{aligned}$$

we write

A.45 $$\begin{aligned} a_{2}^{\infty }(0) = a_{1}^{\infty }(0)\phi \, \frac{\beta }{1!}\, . \end{aligned}$$

For ${{compartment}\, i=3}$, we have

A.46a $$\begin{aligned} \frac{da_{3}^{\infty }(t_{\infty })}{dt_{\infty }}&= k a_{2}^{\infty }(t_{\infty }) - k a_{3}^{\infty }(t_{\infty }), \nonumber \\ \;\; \Rightarrow \;\; s A_{3}^{\infty }(s) -a_{3}^{\infty }(0)&= k A_{2}^{\infty }(s) -k A_{3}^{\infty }(s), \nonumber \\ \;\; \Rightarrow \;\; A_{3}^{\infty }(s)&= \frac{a_{3}^{\infty }(0) + k A_{2}^{\infty }(s)}{s+k}, \nonumber \\&= \frac{a_{3}^{\infty }(0)}{s+k} + \frac{k a_{2}^{\infty }(0)}{(s+k)^{2}} + \frac{k^{2} a_{1}^{\infty }(0)}{(s+k)^{3}}, \end{aligned}$$

A.46b $$\begin{aligned} \;\; \Rightarrow \;\; a_{3}^{\infty }(t_{\infty })&= \left( a_{3}^{\infty }(0) + \frac{k a_{2}^{\infty }(0)}{1!}\, t_{\infty }+ \frac{k^{2} a_{1}^{\infty }(0)}{2!}\, t_{\infty }^{2} \right) e^{-k t_{\infty }}. \end{aligned}$$

To determine $a_{3}^{\infty }(0)$, we use periodicity:

A.46c $$\begin{aligned} a_{3}^{\infty }(0)&= a_{3}^{\infty }(T), \nonumber \\ \;\; \Rightarrow \;\; a_{3}^{\infty }(0)&= a_{3}^{\infty }(0) e^{-k T} + \frac{k a_{2}^{\infty }(0)}{1!} \, T e^{-k T} + \frac{k^{2} a_{1}^{\infty }(0)}{2!} \, T^{2} e^{-k T}, \nonumber \\&= a_{3}^{\infty }(0) e^{-\phi } + \frac{a_{2}^{\infty }(0)}{1!} \, \phi e^{-\phi } + \frac{a_{1}^{\infty }(0)}{2!} \, \phi ^{2} e^{-\phi }, \end{aligned}$$

A.46d $$\begin{aligned} \;\; \Rightarrow \;\; a_{3}^{\infty }(0)&= \beta \times \left( \frac{a_{2}^{\infty }(0)}{1!}\, \phi + \frac{a_{1}^{\infty }(0)}{2!}\, \phi ^{2}\right) \end{aligned}$$

A.46e $$\begin{aligned}&= a_{1}^{\infty }(0) \phi ^{2} \left[ \frac{1}{1!1!} \beta ^{2} + \frac{1}{2!}\beta \right] , \qquad \text {(using (A.45))} . \end{aligned}$$

For ${{compartment}\, i=4}$, we have

A.47a $$\begin{aligned}&\frac{da_{4}^{\infty }(t_{\infty })}{dt_{\infty }} = k a_{3}^{\infty }(t_{\infty }) - k a_{4}^{\infty }(t_{\infty }), \nonumber \\&\qquad \;\; \Rightarrow \;\; s A_{4}^{\infty }(s) -a_{4}^{\infty }(0) = k A_{3}^{\infty }(s) -k A_{4}^{\infty }(s), \nonumber \\&\qquad \;\; \Rightarrow \;\; A_{4}^{\infty }(s) = \frac{a_{4}^{\infty }(0) + k A_{3}^{\infty }(s)}{s+k}, \nonumber \\&\quad = \frac{a_{4}^{\infty }(0)}{s+k} + \frac{k a_{3}^{\infty }(0)}{(s+k)^{2}} + \frac{k^{2} a_{2}^{\infty }(0)}{(s+k)^{3}} + \frac{k^{3} a_{1}^{\infty }(0)}{(s+k)^{4}}, \end{aligned}$$

A.47b $$\begin{aligned}&\qquad \;\; \Rightarrow \;\; a_{4}^{\infty }(t_{\infty }) = \left( a_{4}^{\infty }(0) + \frac{k a_{3}^{\infty }(0)}{1!}\, t_{\infty }\right. \nonumber \\&\left. \qquad + \frac{k^{2} a_{2}^{\infty }(0)}{2!}\, t_{\infty }^{2} + \frac{k^{3} a_{1}^{\infty }(0)}{3!}\, t_{\infty }^{3} \right) e^{-k t_{\infty }}. \end{aligned}$$

To determine $a_{4}^{\infty }(0)$, we use periodicity:

A.47c $$\begin{aligned} a_{4}^{\infty }(0)&= a_{4}^{\infty }(T), \nonumber \\ \;\; \Rightarrow \;\; a_{4}^{\infty }(0)&= a_{4}^{\infty }(0) e^{-k T} + \frac{k a_{3}^{\infty }(0)}{1!} \, T e^{-k T}\nonumber \\&\quad + \frac{k^{2} a_{2}^{\infty }(0)}{2!} \, T^{2} e^{-k T} + \frac{k^{3} a_{1}^{\infty }(0)}{3!} \, T^{3} e^{-k T}, \nonumber \\&= a_{4}^{\infty }(0) e^{-\phi } + \frac{a_{3}^{\infty }(0)}{1!} \, \phi e^{-\phi }\nonumber \\&\quad + \frac{a_{2}^{\infty }(0)}{2!} \, \phi ^{2} e^{-\phi } + \frac{a_{1}^{\infty }(0)}{3!} \, \phi ^{3} e^{-\phi }, \end{aligned}$$

A.47d $$\begin{aligned} \;\; \Rightarrow \;\; a_{4}^{\infty }(0)&= \beta \times \left( \frac{a_{3}^{\infty }(0)}{1!}\, \phi \right. \nonumber \\&\left. \quad + \frac{a_{2}^{\infty }(0)}{2!}\, \phi ^{2} + \frac{a_{1}^{\infty }(0)}{3!}\, \phi ^{2}\right) \end{aligned}$$

A.47e $$\begin{aligned}&= a_{1}^{\infty }(0) \phi ^{3} \left[ \frac{1}{1!1!1!} \beta ^{3} + \left( \frac{1}{1!2!}+\frac{1}{2!1!}\right) \beta ^{2} + \frac{1}{3!}\beta \right] , \qquad \text {(using (A.45) and (A.46e))} . \end{aligned}$$

For ${{compartment}\, i=5}$, we have

A.48a $$\begin{aligned}&\frac{da_{5}^{\infty }(t_{\infty })}{dt_{\infty }} = k a_{4}^{\infty }(t_{\infty }) - k a_{5}^{\infty }(t_{\infty }), \nonumber \\&\qquad \;\; \Rightarrow \;\; s A_{5}^{\infty }(s) -a_{5}^{\infty }(0) = k A_{4}^{\infty }(s) -k A_{5}^{\infty }(s), \nonumber \\&\qquad \;\; \Rightarrow \;\; A_{5}^{\infty }(s) = \frac{a_{5}^{\infty }(0) + k A_{4}^{\infty }(s)}{s+k}, \nonumber \\&\quad = \frac{a_{5}^{\infty }(0)}{s+k} + \frac{k a_{4}^{\infty }(0)}{(s+k)^{2}}\nonumber \\&\qquad + \frac{k^{2} a_{3}^{\infty }(0)}{(s+k)^{3}} + \frac{k^{3} a_{2}^{\infty }(0)}{(s+k)^{4}} + \frac{k^{4} a_{1}^{\infty }(0)}{(s+k)^{5}}, \end{aligned}$$

A.48b $$\begin{aligned}&\qquad \;\; \Rightarrow \;\; a_{5}^{\infty }(t_{\infty }) = \left( a_{5}^{\infty }(0) + \frac{k a_{4}^{\infty }(0)}{1!}\, t_{\infty }+ \frac{k^{2} a_{3}^{\infty }(0)}{2!}\, t_{\infty }^{2} + \frac{k^{3} a_{2}^{\infty }(0)}{3!}\, t_{\infty }^{3}\right. \nonumber \\&\left. \qquad + \frac{k^{4} a_{1}^{\infty }(0)}{3!}\, t_{\infty }^{4} \right) e^{-k t_{\infty }}. \end{aligned}$$

To determine $a_{5}^{\infty }(0)$, we use periodicity:

A.48c $$\begin{aligned} a_{5}^{\infty }(0)&= a_{5}^{\infty }(T), \nonumber \\ \;\; \Rightarrow \;\; a_{5}^{\infty }(0)&= a_{5}^{\infty }(0) e^{-k T} + \frac{k a_{4}^{\infty }(0)}{1!} \, T e^{-k T} + \frac{k^{2} a_{3}^{\infty }(0)}{2!} \, T^{2} e^{-k T}\nonumber \\&\quad + \frac{k^{3} a_{2}^{\infty }(0)}{3!} \, T^{3} e^{-k T} + \frac{k^{3} a_{1}^{\infty }(0)}{4!} \, T^{4} e^{-k T}, \nonumber \\&= a_{5}^{\infty }(0) e^{-\phi } + \frac{a_{4}^{\infty }(0)}{1!} \, \phi e^{-\phi }\nonumber \\&\quad + \frac{a_{3}^{\infty }(0)}{2!} \, \phi ^{2} e^{-\phi } + \frac{a_{2}^{\infty }(0)}{3!} \, \phi ^{3} e^{-\phi } + \frac{a_{1}^{\infty }(0)}{4!} \, \phi ^{4} e^{-\phi }, \end{aligned}$$

A.48d $$\begin{aligned} \;\; \Rightarrow \;\; a_{5}^{\infty }(0)&= \beta \times \left( \frac{a_{4}^{\infty }(0)}{1!}\, \phi + \frac{a_{3}^{\infty }(0)}{2!}\, \phi ^{2} + \frac{a_{2}^{\infty }(0)}{3!}\, \phi ^{3} + \frac{a_{1}^{\infty }(0)}{4!}\, \phi ^{4}\right) \end{aligned}$$

A.48e $$\begin{aligned}&= a_{1}^{\infty }(0) \phi ^{4} \left[ \frac{1}{1!1!1!1!} \beta ^{4} + \left( \frac{1}{1!1!2!}+\frac{1}{1!2!1!}+\frac{1}{2!1!1!}\right) \beta ^{3}\right. \nonumber \\&\quad \left. + \left( \frac{1}{1!3!}+\frac{1}{2!2!}+\frac{1}{3!1!}\right) \beta ^{2}\right. \nonumber \\&\left. \quad + \frac{1}{4!}\beta \right] , \nonumber \\&\qquad \text {(using } (A.45),(A.46e), (A.47e)) . \end{aligned}$$

The process continues, and clearly, the solution for the *n*th transit compartment at steady-state is given by

$$\begin{aligned} a_{i}^{\infty }(t_{\infty })= & {} \left[ a_{i}^{\infty }(0) + \frac{a_{i-1}^{\infty }(0)}{1!}(kt_{\infty }) + \frac{a_{i-2}^{\infty }(0)}{2!}(kt_{\infty })^{2}\right. \\&\left. + \cdots + \frac{a_{1}^{\infty }(0)}{(i-1)!}(kt_{\infty })^{i-1} \right] e^{-k t_{\infty }}, \end{aligned}$$

or

A.49a $$\begin{aligned} \displaystyle {\boxed {a_{i}^{\infty }(t_{\infty }) = \left( \sum _{p=0}^{i-1} \frac{a_{i-p}^{\infty }(0)}{p!} (kt_{\infty })^{p} \right) e^{-k t_{\infty }} }}, \qquad \text {for } i=1, \ldots , n. \end{aligned}$$

The coefficients $a_{i}(0)$ (the steady-state dosing interval initial values) may be found using

A.49b $$\begin{aligned} \boxed {a_{1}^{\infty }(0) = \frac{FD_{0}}{1-e^{-\phi }}}. \end{aligned}$$

together with the recurrence relation (for $i=2, \ldots , n$)

A.49c $$\begin{aligned} \boxed {a_{i}(0) = \beta \times \left( \frac{a_{i-1}^{\infty }(0)}{1!}\, \phi + \frac{a_{i-2}^{\infty }(0)}{2!}\, \phi ^{2} + \frac{a_{i-3}^{\infty }(0)}{3!}\, \phi ^{3} + \cdots + \frac{a_{1}^{\infty }(0)}{(i-1)!}\, \phi ^{i-1}\right) \;\; = \;\; \beta \sum _{p=1}^{i-1} \frac{a_{i-p}^{\infty }(0)}{p!}\phi ^{p}}. \end{aligned}$$

The recurrence relation is clear when considering the generalisation of (A.46d), (A.47d), (A.48d), etc. A closed-form expression for $a_{i}(0)$ is also possible by considering the generalisation of (A.46e), (A.47e), (A.48e), etc. The coefficient of $\beta ^{K}$ in the expression for $a_{i}^{\infty }(0)$ is given by a reciprocal factorial product related to partitioning and permutations of a set of *K* positive integers which sum to $(i-1)$. These coefficients may be written in terms of the Stirling number of the second kind [40], *S*, given by

A.49d $$\begin{aligned} \boxed {S(n,q)=\frac{1}{q!}\sum _{p=0}^{q} (-1)^{p}\, \begin{pmatrix} q \\ p \end{pmatrix} (q-p)^{n}}, \qquad \text {where} \quad \begin{pmatrix} q \\ p \end{pmatrix}=\frac{q!}{p!(q-p)!} \text { is the binomial coefficient}, \end{aligned}$$

and $S(0,0)=1$. Rewriting (A.42), (A.45), (A.46e), (A.47e), (A.48e) so that the bracketed polynomials in $\beta$ have integer coefficients, and also continuing the process up to the 10${\text {th}}$ transit compartment, we find that:

$$\begin{aligned} a_{1}^{\infty }(0)&= \frac{FD_{0}}{1-e^{-\phi }} , \\ a_{2}^{\infty }(0)&= \frac{FD_{0}}{1-e^{-\phi }}\, \phi \beta , \\ a_{3}^{\infty }(0)&= \frac{FD_{0}}{1-e^{-\phi }}\, \frac{\phi ^{2}}{2!} \left( 2\beta ^{2}+\beta \right) , \\ a_{4}^{\infty }(0)&= \frac{FD_{0}}{1-e^{-\phi }}\, \frac{\phi ^{3}}{3!} \left( 6\beta ^{3}+6\beta ^{2}+\beta \right) , \\ a_{5}^{\infty }(0)&= \frac{FD_{0}}{1-e^{-\phi }}\, \frac{\phi ^{4}}{4!} \left( 24\beta ^{4}+36\beta ^{3}+14\beta ^{2}+\beta \right) , \\ a_{6}^{\infty }(0)&= \frac{FD_{0}}{1-e^{-\phi }}\, \frac{\phi ^{5}}{5!} \left( 120\beta ^{5}+240\beta ^{4}+150\beta ^{3}+30\beta ^{2}+\beta \right) , \\ a_{7}^{\infty }(0)&= \frac{FD_{0}}{1-e^{-\phi }}\, \frac{\phi ^{6}}{6!} \left( 720\beta ^{6}+1800\beta ^{5}\right. \\&\quad \left. +1560\beta ^{4}+540\beta ^{3}+62\beta ^{2}+\beta \right) , \\ a_{8}^{\infty }(0)&= \frac{FD_{0}}{1-e^{-\phi }}\, \frac{\phi ^{7}}{7!} \left( 5040\beta ^{7}\right. \\&\quad \left. +15120\beta ^{6}+16800\beta ^{5}+8400\beta ^{4}+1806\beta ^{3}+126\beta ^{2}+\beta \right) , \\ a_{9}^{\infty }(0)&= \frac{FD_{0}}{1-e^{-\phi }}\, \frac{\phi ^{8}}{8!} \left( 40320\beta ^{8}+141120\beta ^{7}\right. \\&\quad \left. +191520\beta ^{6}+126000\beta ^{5}+40824\beta ^{4}+5796\beta ^{3}+254\beta ^{2}+\beta \right) , \\ a_{10}^{\infty }(0)&= \frac{FD_{0}}{1-e^{-\phi }}\, \frac{\phi ^{9}}{9!} \left( 362880\beta ^{9}\right. \\&\quad \left. +1451520\beta ^{8}+2328480\beta ^{7}+1905120\beta ^{6}+834120\beta ^{5} \right. \\&\quad \left. + 186480\beta ^{4}+18150\beta ^{3}+510\beta ^{2}+\beta \right) . \end{aligned}$$

In terms of the Stirling number of the second kind, the closed-form expression for $a_{i}^{\infty }(0)$ is

A.49e $$\begin{aligned} \boxed {a_{i}^{\infty }(0) = \frac{FD_{0}}{1-e^{-\phi }}\, \frac{\phi ^{i-1}}{(i-1)!} \sum _{p=0}^{i-1} p! S(i-1,p)\beta ^{p}}, \qquad \text {for } i=1, \ldots , n. \end{aligned}$$

For the absorption compartment, we have

A.50a $$\begin{aligned} \frac{da_{b}^{\infty }(t_{\infty })}{dt_{\infty }}&= k a_{n}^{\infty }(t_{\infty }) - k_{a} a_{b}^{\infty }(t_{\infty }), \nonumber \\ \;\; \Rightarrow \;\; s A_{b}^{\infty }(s) -a_{b}^{\infty }(0)&= k A_{n}^{\infty }(s) -k_{a} A_{b}^{\infty }(s), \nonumber \\ \;\; \Rightarrow \;\; A_{b}^{\infty }(s)&= \frac{a_{b}^{\infty }(0) + k A_{n}^{\infty }(s)}{s+k_{a}}, \nonumber \\&= \frac{a_{b}^{\infty }(0)}{s+k_{a}} + \frac{k}{s+k_{a}} \sum _{p=1}^{n} \frac{k^{n-p}a_{p}^{\infty }(0)}{(s+k)^{n-p+1}}, \end{aligned}$$

A.50b $$\begin{aligned} \;\; \Rightarrow \;\; a_{b}^{\infty }(t_{\infty })&= a_{b}^{\infty }(0)e^{-k_{a}t_{\infty }}\nonumber \\&\quad + {\mathcal {L}}^{-1}\left\{ \frac{k}{s+k_{a}} \sum _{p=1}^{n} \frac{k^{n-p}a_{p}^{\infty }(0)}{(s+k)^{n-p+1}} \right\} . \end{aligned}$$

Now, since

A.50c $$\begin{aligned}&{\mathcal {L}}^{-1}\left\{ \frac{1}{(s+k_{a})(s+k)^{n-p+1}} \right\} \nonumber \\&\quad = \frac{1}{(n-p)!(k-k_{a})^{n-p+1}} e^{-k_{a}t_{\infty }}\, \gamma (n-p+1,(k-k_{a})t_{\infty }), \end{aligned}$$

we find that

A.50d $$\begin{aligned} \boxed {a_{b}^{\infty }(t_{\infty }) = \left[ a_{b}^{\infty }(0) + \sum _{p=1}^{n} \frac{a_{p}^{\infty }(0)}{(n-p)!} \left( \frac{k}{k-k_{a}}\right) ^{n-p+1} \, \gamma (n-p+1,(k-k_{a})t_{\infty }) \right] e^{-k_{a}t_{\infty }}}. \end{aligned}$$

To determine $a_{b}^{\infty }(0)$, we use periodicity:

A.50e $$\begin{aligned} a_{b}^{\infty }(0)&= a_{b}^{\infty }(T), \nonumber \\ \;\; \Rightarrow \;\; a_{b}^{\infty }(0)&= \left[ a_{b}^{\infty }(0) + \sum _{p=1}^{n} \frac{a_{p}^{\infty }(0)}{(n-p)!} \left( \frac{k}{k-k_{a}}\right) ^{n-p+1} \, \gamma (n-p+1,(k-k_{a})T) \right] e^{-k_{a}T}, \end{aligned}$$

so that

A.50f $$\begin{aligned} \boxed {a_{b}^{\infty }(0) = \beta _{a}\sum _{p=1}^{n} \frac{a_{p}^{\infty }(0)}{(n-p)!} \left( \frac{k}{k-k_{a}}\right) ^{n-p+1} \, \gamma (n-p+1,(k-k_{a})T) } , \end{aligned}$$

where

A.50g $$\begin{aligned} \boxed {\beta _{a} = \frac{e^{-k_{a}T}}{1-e^{-k_{a}T}}}. \end{aligned}$$

Finally, for the central compartment, we have

A.51a $$\begin{aligned}&\frac{da_{c}^{\infty }(t_{\infty })}{dt_{\infty }} = k_{a} a_{b}^{\infty }(t_{\infty }) - k_{e}a_{c}^{\infty }(t_{\infty }), \nonumber \\&\qquad \;\; \Rightarrow \;\; s A_{c}^{\infty }(s) -a_{c}^{\infty }(0) = k_{a} A_{b}^{\infty }(s) - k_{e}A_{c}^{\infty }(s), \nonumber \\&\quad \;\; \Rightarrow \;\; A_{c}^{\infty }(s) = \frac{a_{c}^{\infty }(0) + k_{a} A_{b}^{\infty }(s)}{s+k_{e}}, \nonumber \\&\qquad = \frac{a_{c}^{\infty }(0)}{s+k_{e}} + \frac{k_{a}}{s+k_{e}} \left( \frac{a_{b}^{\infty }(0)}{s+k_{a}}\right. \nonumber \\&\qquad \left. + \frac{k}{s+k_{a}} \sum _{p=1}^{n} \frac{k^{n-p}a_{p}^{\infty }(0)}{(s+k)^{n-p+1}}\right) , \end{aligned}$$

A.51b $$\begin{aligned}&\;\; \Rightarrow \;\; a_{c}^{\infty }(t_{\infty }) = \frac{a_{c}^{\infty }(0)}{s+k_{e}} + \frac{k_{a}}{(s+k_{e})(s+k_{a})} \left( a_{b}^{\infty }(0)\right. \nonumber \\&\qquad \left. + k \sum _{p=1}^{n} \frac{k^{n-p}a_{p}^{\infty }(0)}{(s+k)^{n-p+1}}\right) . \end{aligned}$$

Now, since

A.51c $$\begin{aligned} {\mathcal {L}}^{-1} \left\{ \frac{1}{(s+k_{a})(s+k_{e})}\right\} = \frac{H(t_{\infty })}{k_{e}-k_{a}} \left( e^{-k_{a}t_{\infty }}-e^{-k_{e}t_{\infty }}\right) , \end{aligned}$$

and

A.51d $$\begin{aligned}&{\mathcal {L}}^{-1} \left\{ \frac{1}{(s+k_{a})(s+k_{e})(s+k)^{n-p+1}}\right\} \nonumber \\&\quad = \frac{1}{(n-p)!(k_{a}-k_{e})}\left( \frac{e^{-k_{e}t_{\infty }}}{(k-k_{e})^{n-p+1}} \, \gamma (n-p+1,(k-k_{e})t_{\infty }) \;\right. \nonumber \\&\qquad \left. - \; \frac{e^{-k_{a} t_{\infty }}}{(k-k_{a})^{n-p+1}} \, \gamma (n-p+1,(k-k_{a})t_{\infty }) \right) , \end{aligned}$$

we find that

A.51e $$\begin{aligned} \boxed { \begin{aligned} a_{c}^{\infty }(t_{\infty })&= a_{c}^{\infty }(0)e^{-k_{e}t_{\infty }} + \frac{k_{a}}{k_{a}-k_{e}} \times \Bigg \{ a_{b}^{\infty }(0)(e^{-k_{e}t_{\infty }}-e^{-k_{a}t_{\infty }}) \; + \; \Bigg . \\&\; \sum _{p=1}^{n} \frac{a_{p}^{\infty }(0)}{(n-p)!} \left[ \left( \frac{k}{k-k_{e}}\right) ^{n-p+1} \, e^{-k_{e}t_{\infty }} \, \gamma (n-p+1,(k-k_{e})t_{\infty }) \; - \; \right. \\&\qquad \qquad \qquad \left. \left( \frac{k}{k-k_{a}}\right) ^{n-p+1} \, e^{-k_{a} t_{\infty }} \, \gamma (n-p+1,(k-k_{a})t_{\infty }) \right] \Bigg . \Bigg \} . \end{aligned} } \end{aligned}$$

To determine $a_{c}^{\infty }(0)$, we use periodicity:

A.51f $$\begin{aligned} \begin{aligned} a_{c}^{\infty }(0)&= a_{c}^{\infty }(T), \\ \;\; \Rightarrow \;\; a_{c}^{\infty }(0)&= a_{c}^{\infty }(0)e^{-k_{e}T} + \frac{k_{a}}{k_{a}-k_{e}}\\&\quad \times \Bigg \{ a_{b}^{\infty }(0)(e^{-k_{e}T}-e^{-k_{a}T}) \; + \; \Bigg . \\&\quad \sum _{p=1}^{n} \frac{a_{p}^{\infty }(0)}{(n-p)!} \left[ \left( \frac{k}{k-k_{e}}\right) ^{n-p+1} \,\right. \\&\quad \left. e^{-k_{e}T} \, \gamma (n-p+1,(k-k_{e})T) \; - \; \right. \\&\quad \left. \left( \frac{k}{k-k_{a}}\right) ^{n-p+1} \,\right. \\&\quad \left. e^{-k_{a} T} \, \gamma (n-p+1,(k-k_{a})T) \right] \Bigg . \Bigg \} , \end{aligned} \end{aligned}$$

so that

A.51g $$\begin{aligned} \boxed { \begin{aligned} a_{c}^{\infty }(0)&= \beta _{c} \times \Bigg \{ a_{b}^{\infty }(0)(e^{-k_{e}T}-e^{-k_{a}T}) \; + \; \Bigg . \\&\; \sum _{p=1}^{n} \frac{a_{p}^{\infty }(0)}{(n-p)!} \left[ \left( \frac{k}{k-k_{e}}\right) ^{n-p+1} \, e^{-k_{e}T} \, \gamma (n-p+1,(k-k_{e})T) \; - \; \right. \\&\qquad \qquad \qquad \left. \left( \frac{k}{k-k_{a}}\right) ^{n-p+1} \, e^{-k_{a} T} \, \gamma (n-p+1,(k-k_{a})T) \right] \Bigg . \Bigg \} , \end{aligned} } \end{aligned}$$

where

A.51h $$\begin{aligned} \boxed {\beta _{c} = \frac{k_{a}}{(k_{a}-k_{e})(1-e^{-k_{e}T})}}. \end{aligned}$$

#### Computational evaluation of the lower incomplete gamma function

Our preferred method for evaluating the lower incomplete gamma function is using the built-in function gammainc in MATLAB, which computes the normalised function $\displaystyle {P(n,t)=\frac{\gamma (n,t)}{\varGamma (n)}}$. In Fig. 16, we show a typical result comparing run times and computed values of $\gamma (n,t)$ for a given *t* and range of *n*, for five different methods. For computations in MATLAB [35], it is clear that the quickest method is using the built-in command gammainc. All methods give values in agreement, except for the symbolic computation of the sum in (3.17) for large *n*. This is due to rounding and truncation errors introduced when evaluating computed symbolic expressions. Hard-coding the expressions in this sum results in the second shortest run time, but this method is not practical for $n>10$. Use of the log-gamma function is an order of magnitude slower, but is still much faster than employing a numerical integration method. We conclude that built-in functions, either evaluating $\gamma (n,t)$ directly or via the log-gamma and distribution functions, represent a practical approach to evaluating $\gamma (n,t)$. More efficient methods involving series and continued fraction expansions may be investigated [1, 2, 17, 41, 50], but are beyond our scope here.

## Appendix 2: time course results

### Single continuous infusion as limit of IV equi-bolus dosing

Recalling the IV equi-dosing result (3.2), we consider dosing regimens which keep the ratio $\frac{D_{0}}{T}$ fixed, and let $T\rightarrow 0$. That is, consider a fixed dosing rate $R = \frac{D_{0}}{T}$. Now consider the drug level at the end of the *M*th dosing interval:

$$\begin{aligned} a_{c}^{M}(T) \;\; = \;\; FD_{0}\frac{1-e^{-Mk_{e}T}}{1-e^{-k_{e}T}} \, e^{-k_{e}T} \;\; = \;\; F R \, T \, \frac{1-e^{-Mk_{e}T}}{1-e^{-k_{e}T}} \, e^{-k_{e}T}. \end{aligned}$$

Suppose that by the end of the *M*th dosing interval the total time since the first dose is $t_{end}$ (fixed constant). Then $t_{end}=MT$, and so

$$\begin{aligned} a_{c}(t_{end}) \;\; = \;\; a_{c}^{M}(T) \;\; = \;\; F R \, \frac{1-e^{-k_{e}t_{end}}}{1-e^{-k_{e}T}} \, T e^{-k_{e}T}. \end{aligned}$$

Then taking infinitesimally small dosing interval (and so an infinitesimal dose), our result becomes

$$\begin{aligned} a_{c}(t_{end}) \rightarrow \lim _{T\rightarrow 0} FR (1-e^{-k_{e}t_{end}}) \frac{T e^{-k_{e}T}}{1-e^{-k_{e}T}} \, . \end{aligned}$$

Now, using l’Hopital’s Rule, we see that

$$\begin{aligned} a_{c}(t_{end}) \rightarrow FR (1-e^{-k_{e}t_{end}}) \lim _{T\rightarrow 0} \frac{e^{-k_{e}T}(1-k_{e}T)}{k_{e}e^{-k_{e}T}} \, . \end{aligned}$$

Thus, as $T \rightarrow 0$, we have

$$\begin{aligned} a_{c}(t_{end}) \rightarrow FR (1-k_{e}t_{end}) \times \frac{1}{k_{e}} \;\; = \;\; \frac{FR}{k_{e}}(1-e^{-k_{e}t_{end}}). \end{aligned}$$

Now, if $R=\frac{D_{0}}{T}=k_{in}$, then

B.1 $$\begin{aligned} a_{c}(t_{end}) \rightarrow \frac{Fk_{in}}{k_{e}} (1-e^{-k_{e}t_{end}}), \end{aligned}$$

which is equivalent to the continuous infusion solution (4.1). $\blacksquare$

### Two-compartment oral equi-bolus dosing

Here, we note some properties of the two-compartment equi-bolus dosing solutions (3.10) and (3.11). Firstly, the minimum drug level on the *n*th dosing interval is given by $a_{c}^{n}(0)$, while for a local maximum (a **peak** level) in the time course for $a_{c}^{n}(t_{n})$ at $t_{n}=t^{*}_{n}$, we need $a_{c}^{n}{}\, '(t^{*}_{n})=0$. We find, by using (3.10), that:

B.2 $$\begin{aligned} t^{*}_{n} \;\; = \;\; \frac{1}{k_{a}-k_{e}} \; \log \left( \, \frac{k_{a}}{k_{e}} \, \frac{1-e^{-nk_{a}T}}{1-e^{-k_{a}T}}\, \frac{1-e^{-k_{e}T}}{1-e^{-nk_{e}T}}\, \right) \; . \end{aligned}$$

If $0<t^{*}_{n}<T$, then there is a peak blood drug level in the $n{\text {th}}$ dosing interval, given by

B.3 $$\begin{aligned}&a_{c}^{n}{}_{,max} \;\; = \;\; a_{c}^{n}\left( t^{*}_{n}\right) \nonumber \\&\quad \;\; = \;\; FD_{0}\left\{ \left( \frac{k_{e}}{k_{a}}\right) ^{k_{e}} \; \left( \frac{1-e^{-k_{a}T}}{1-e^{-nk_{a}T}}\right) ^{k_{e}} \; \left( \frac{1-e^{-nk_{e}T}}{1-e^{-k_{e}T}}\right) ^{k_{a}} \; \right\} ^{\frac{1}{k_{a}-k_{e}}} \; . \end{aligned}$$

So we may write the maximum and minimum values of $a_{c}^{n}$ on [0, *T*] as:

B.4 $$\begin{aligned} a_{c}^{n}{}_{,min}&= a_{c}^{n}(0) \;\; = \;\; FD_{0}\; \frac{k_{a}}{k_{a}-k_{e}} \; \left\{ \, \frac{1-e^{-nk_{e}T}}{1-e^{-k_{e}T}} \; - \; \frac{1-e^{-nk_{a}T}}{1-e^{-k_{a}T}} \, \right\} \; , \end{aligned}$$

B.5 $$\begin{aligned} a_{c}^{n}{}_{,max}&= a_{c}^{n}\left( \min (t^{*}_{n},T)\right) \, , \;\;\; \text {(using ({B}.2))} \; . \end{aligned}$$

At steady-state, a local maximum in the time course is ensured, occurring at

B.6 $$\begin{aligned} t^{*}_{\infty } = \frac{1}{k_{a}-k_{e}} \; \log \left( \, \frac{k_{a}}{k_{e}} \, \frac{1-e^{-k_{e}T}}{1-e^{-k_{a}T}}\, \right) . \end{aligned}$$

The steady-state minimum and maximum drug levels are then given by

B.7 $$\begin{aligned} a_{c}^{\infty }{}_{,min}&= a_{c}^{\infty }(0) \;\; = \;\; FD_{0}\; \frac{k_{a}}{k_{a}-k_{e}} \; \left\{ \, \frac{1}{1-e^{-k_{e}T}} \; - \; \frac{1}{1-e^{-k_{a}T}} \, \right\} \; , \end{aligned}$$

B.8 $$\begin{aligned} a_{c}^{\infty }{}_{,max}&= a_{c}^{\infty }\left( t^{*}_{\infty }\right) \;\; = \;\; FD_{0}\left\{ \left( \, \frac{k_{e}}{k_{a}}\right) ^{k_{e}} \; \frac{ \left( 1-e^{-k_{a}T} \right) ^{k_{e}} }{ \left( 1-e^{-k_{e}T} \right) ^{k_{a}} } \, \right\} ^{\frac{1}{k_{a}-k_{e}}} \; . \end{aligned}$$

### Transit compartment parameter identifiability

In Fig. 17, we show time courses for a transit compartment model with $n=1$ with a single bolus dose. Non-identifiability of *k* and $k_{a}$ is clear, due to the flip-flop phenomenon discussed in ‘Transit compartments—smoothed delays, lag time and data fitting (single-dose)’. For our transit compartment model, this problem is unique to $n=1$, and no such issues are seen with $n>1$. In Fig. 18, we plot a typical parameter estimation result for $n=10$ compartments, using the method described in ‘Transit compartments—smoothed delays, lag time and data fitting (single-dose)’. With “perfect” pseudo-data, we see the base parameter set being recovered exactly, and with noisy pseudo-data, the parameter estimates are in good agreement with the base set. A formal structural and practical identifiability analysis is proposed as future work, and will use transfer functions and other methods described in [13].

### Transit compartment equi-bolus dosing

In Figs. 19–20, we show time course results for a transit compartment model under multi-dosing input (Mt,Beq), using parameters taken from the fitted data in Fig. 5. Each figure shows the analytical results for drug levels in a number of transit compartments, with $n=10$ transit compartments in total. The absorption and central compartments are also shown. For validation, results from a numerical ODE solver are also shown. For tracking the approach to steady-state, we mark the steady-state profile and dosing interval average for each compartment plotted. For each of the transit compartments, the steady-state dosing interval average may be computed, for example, from (2.6) by integrating each of the compartment ODEs in turn. We find the transit compartment averages to be

B.9 $$\begin{aligned} \overline{a_{i}^{\infty }}_{[0,T]} = \frac{FD_{0}}{kT}, \qquad \text {for } i=1, \ldots , n \;. \end{aligned}$$

Similarly, for the absorption and central compartments, we find

B.10 $$\begin{aligned} \overline{a_{b}^{\infty }}_{[0,T]} = \frac{FD_{0}}{k_{a}T}, \qquad \overline{a_{c}^{\infty }}_{[0,T]} = \frac{FD_{0}}{k_{e}T}. \end{aligned}$$

In both figures, we observe the delay effect, as the peak drug levels appear later further through the transit cascade. In Fig. 19, the transit rate constant *k* is taken from the earlier data fitting, and the system almost reaches steady-state by the second dosing interval. In Fig. 20, we take *k* four-fold lower, which increases the smoothed delay, and hence delays the approach to steady-state. In this case, the delay effect is more evident throughout the transit cascade.

## Appendix 3: equi-dosing regimen region for IV equi-bolus dosing with loading dose

In Fig. 21, we show a computed example EDRR for the three-parameter $(T,D_{0},D_{L})$ regimen including loading dose. Cross sections of fixed $D_{L}$ through the EDRR are chopped petals, while the EDRR shadow in the $(T,D_{0})$-plane is the full petal EDRR for the steady-state.
